# Genetic determinants of common epilepsies: a meta-analysis of genome-wide association studies

**DOI:** 10.1016/S1474-4422(14)70171-1

**Published:** 2014-09-01

**Authors:** International League Against Epilepsy Consortium on Complex Epilepsies

## Abstract

**Background:**

The epilepsies are a clinically heterogeneous group of neurological disorders. Despite strong evidence for heritability, genome-wide association studies have had little success in identification of risk loci associated with epilepsy, probably because of relatively small sample sizes and insufficient power. We aimed to identify risk loci through meta-analyses of genome-wide association studies for all epilepsy and the two largest clinical subtypes (genetic generalised epilepsy and focal epilepsy).

**Methods:**

We combined genome-wide association data from 12 cohorts of individuals with epilepsy and controls from population-based datasets. Controls were ethnically matched with cases. We phenotyped individuals with epilepsy into categories of genetic generalised epilepsy, focal epilepsy, or unclassified epilepsy. After standardised filtering for quality control and imputation to account for different genotyping platforms across sites, investigators at each site conducted a linear mixed-model association analysis for each dataset. Combining summary statistics, we conducted fixed-effects meta-analyses of all epilepsy, focal epilepsy, and genetic generalised epilepsy. We set the genome-wide significance threshold at p<1·66 × 10^−8^.

**Findings:**

We included 8696 cases and 26 157 controls in our analysis. Meta-analysis of the all-epilepsy cohort identified loci at 2q24.3 (p=8·71 × 10^−10^), implicating *SCN1A*, and at 4p15.1 (p=5·44 × 10^−9^), harbouring *PCDH7*, which encodes a protocadherin molecule not previously implicated in epilepsy. For the cohort of genetic generalised epilepsy, we noted a single signal at 2p16.1 (p=9·99 × 10^−9^), implicating *VRK2* or *FANCL*. No single nucleotide polymorphism achieved genome-wide significance for focal epilepsy.

**Interpretation:**

This meta-analysis describes a new locus not previously implicated in epilepsy and provides further evidence about the genetic architecture of these disorders, with the ultimate aim of assisting in disease classification and prognosis. The data suggest that specific loci can act pleiotropically raising risk for epilepsy broadly, or can have effects limited to a specific epilepsy subtype. Future genetic analyses might benefit from both lumping (ie, grouping of epilepsy types together) or splitting (ie, analysis of specific clinical subtypes).

**Funding:**

International League Against Epilepsy and multiple governmental and philanthropic agencies.

## Introduction

Epilepsy is a common disorder, affecting up to 4% of people at some time in life.^1^ The disorder includes a group of heterogeneous syndromes defined by clinical, electroencephalographic (EEG), and brain imaging criteria.^2^ Broadly, the epilepsies are divided clinically into generalised and focal forms. Genetic factors contribute to both, as shown by findings from familial aggregation and twin studies.^3^ Causative mutations in many genes, including some genes coding for ion channel subunits and others involved in synaptic function or brain development, have been reported.^3^, ^4^ Most of these findings were reported in patients with fairly rare familial epilepsies segregating in a Mendelian way or epilepsies arising from de-novo mutations (particularly in patients with severe infantile epilepsies).^5^, ^6^, ^7^

The genetic determinants underlying the common epilepsies, for which clinical genetic data suggest complex inheritance, remain largely unknown. Some evidence suggests a role for rare sequence and copy number variants,^8^, ^9^, ^10^ whereas the contribution of common polymorphisms is still unclear,^11^, ^12^ partly as a result of the relatively small sample sizes analysed to date.

Findings from the largest genome-wide association study (GWAS) in epilepsy so far, including 3445 patients with focal epilepsy,^13^ showed no variants of genome-wide significance. More recently, findings from a study of 1018 patients with mesial temporal lobe epilepsy with hippocampal sclerosis (a subtype of focal epilepsy) implicated the 2q24.3 region around the gene encoding the sodium channel SCN1A,^14^ and findings from an independent study of Han Chinese patients with known or suspected lesional focal epilepsy showed evidence for a risk allele at 1q32 on the basis of a discovery sample of 504 cases.^15^

For generalised epilepsy, a GWAS included 1527 European patients with genetic generalised epilepsies in the discovery analysis and 1493 patients in the replication cohort; investigators reported evidence for common risk alleles at 2p16.1 and 17q21.32, and suggestive evidence at the *SCN1A* locus.^16^ Additionally, associations were reported for the juvenile myoclonic subtype of genetic generalised epilepsy at 1q43 and for absence epilepsy at 2q22.3.^16^

In a large multicentre collaboration, we undertook a meta-analysis to detect variants that could increase risk for common epilepsies. In view of clinical evidence that some genetic factors might increase risk for epilepsy broadly and in a syndrome-specific manner,^17^, ^18^, ^19^ we prespecified three analyses as part of the study. Variants were sought that affected risk for all epilepsies, genetic generalised epilepsy (previously known as idiopathic generalised epilepsy),^2^, ^20^ or focal epilepsy.

## Methods

### Study design and participants

We did a meta-analysis of data from 12 previously published or unpublished genetic cohort studies from EPICURE,^16^ EPIGEN,^13^ Philadelphia (PA, USA), the Imperial-Liverpool-Melbourne Collaboration,^21^ GenEpa,^13^ and Hong Kong (China)^15^ (appendix). We identified these studies from the scientific literature (through searches of PubMed in December, 2011, with the terms “epilepsy”, “seizures”, and “association studies”), through publicity via Chapters of the International League Against Epilepsy, and during international conferences. All participants in these 12 case cohorts (and their associated controls) were of European, Asian, or African ancestry (table 1, appendix).

**Table 1:** Cases and controls, by index GWAS

	Ethnic origin*	**All epilepsy (n=8696)**	**Genetic generalised epilepsy (n=2606)**	**Focal epilepsy (n=5310)**	**Population controls**‡**(n=26 157)**
EPIGEN-Dublin	Irish	638	..	520	2232
EPIGEN-Brussels	Belgian	505	48	406	1675
EPIGEN-Duke†	African-American and European-American	760	102	551	504
EPIGEN-London	British and other	1007	93	773	2494
ILM Collaboration	European descent	1703	212	1263	2699
GenEpa	Finnish	422	..	422	1963
EPICURE	Northwest European	1440	1440	..	2454
Philadelphia_550_AA†	African-American	324	81	222	2746
Philadelphia_550_CAU	European-American	819	440	378	5736
Philadelphia_Omni_AA§	African-American	106	..	..	97
Philadelphia_Omni_CAU	European-American	485	190	288	682
Hong Kong	Asian-Han	487	..	487	2875

Numbers of cases and controls are after quality control filtering. GWAS=genome-wide association study. ILM=Imperial-Liverpool-Melbourne.

* Broad ethnic origin of the cohort. Other indicates people of mixed ethnic origin, as would be expected in a cosmopolitan population. European descent refers to white European.

† EPIGEN-Duke individuals of African-American ancestry were merged with participants in the Philadelphia_550_AA cohort.

‡ See appendix for further details about control cohorts.

§ Small sample size prohibited epilepsy subtype analysis in this cohort.

The genetic cohort studies used a combination of population-based datasets as controls. These control cohorts were either screened or unscreened by questionnaire for neurological disorders (table 1, appendix).

All study participants provided written informed consent for DNA analysis. Local institutional review boards reviewed and approved study protocols at each site.

### Procedures

We classified seizures and epilepsy syndromes according to the International League Against Epilepsy terminology.^2^, ^20^ For all cases, epilepsy specialists assessed phenotype at the source centre. Patients with epilepsy were assigned to one of three phenotypic categories: genetic generalised epilepsy, focal epilepsy, or unclassified epilepsy.

Criteria for genetic generalised epilepsy were tonic-clonic, absence, or myoclonic seizures with generalised spike–wave discharges on EEG and no evidence of an acquired cause. In rare instances the criterion for a diagnostic EEG was waived when clear clinical evidence suggested myoclonic or absence seizures with tonic-clonic seizures, and no evidence for an acquired cause. The International League Against Epilepsy has adopted the term genetic generalised epilepsy for syndromes previously known as idiopathic or primary generalised epilepsies, in view of strong evidence for a genetic basis from genetic epidemiological and twin studies and an absence of identified acquired factors.^2^, ^20^

In the phenotypic category of focal epilepsy, we included patients with a confirmed diagnosis of focal epilepsy, including cases with focal structural brain lesions. These cases were predominantly adults, and as such, cases of benign epilepsy of childhood with centro-temporal spikes were not specifically included.

Unclassified epilepsy consisted of patients in whom there was neither electroclinical evidence for generalised epilepsy nor evidence for a focal seizure onset. Additionally, cases with evidence for both generalised and focal epilepsy were included here.

The phenotyping committee curated patient phenotypes into a single database. Details relating to individual case cohorts are provided in the appendix. Analyses were done for three phenotypic groups: genetic generalised epilepsy, focal epilepsy, and all epilepsy (consisting of all patients with a confirmed diagnosis of epilepsy, including genetic generalised epilepsy, focal epilepsy, and unclassified epilepsy).

### Statistical analysis

We used prespecified criteria for quality control to filter cases and controls from the 12 cohorts (appendix). Because contributing sites had used different genotyping platforms, we did imputation to infer genotypes for common genetic variants that were not directly genotyped, allowing us to combine results across sites. Each of the five sites imputed their study datasets according to a standardised protocol. This protocol used IMPUTE2 to infer and impute haplotypes, with the 1000 Genomes Phase I (interim) June, 2011, reference panel (appendix).

Investigators at each site did a linear mixed-model association analysis for each of their datasets with FaSTLMM (version 1.09).^22^ This analysis uses linear regression, including a polygenic term designed to account for the contributions of population stratification and causal variants aside from the one being tested. Although we were assessing a binary trait, we used linear regression (rather than logistic regression) because we expected effect sizes to be small. We did this analysis separately for each of the preselected phenotypic categories of epilepsy (all epilepsy, genetic generalised epilepsy, and focal epilepsy). Sex was included as a covariate.

We did a fixed-effects meta-analysis with METAL (version generic-metal-2011-03-25).^23^ Because almost all epilepsy cases were of European descent (table 1), we chose a fixed-effects model to optimise power. Single nucleotide polymorphisms showing significant amounts of heterogeneity (p<0·05) were removed before application of the fixed-effects analysis. We applied genomic correction to the association analysis results for each dataset before combining for meta-analysis. These steps were done separately for each of the three phenotypic tests.

We set our genome-wide threshold for statistical significance at 1·66 × 10^−8^, representing an empirical Bonferroni correction of the 5 × 10^−8^ genome-wide significance threshold for three tests. We regarded signals with p values between 1·66 × 10^−8^ and 5 × 10^−7^ as suggestive evidence of association.

We calculated the proportion of phenotypic variance a variant must explain (heritability) for the detection power to be at least 80%. We used variance explained on the liability scale,^24^ for which we assumed a point prevalence of 0·5% for all epilepsy, 0·2% for genetic generalised epilepsy, and 0·3% for focal epilepsy.^25^ The required heritability was 0·07% or greater for all epilepsy, 0·17% or greater for genetic generalised epilepsy, and 0·10% or greater for focal epilepsy (appendix).

In addition to the main association analysis, we did logistic regression for variants in a 1 megabase window centred on each variant that showed suggestive evidence of association (p<5 × 10^−7^) from any of the three meta-analyses (all epilepsy, genetic generalised epilepsy, or focal epilepsy). The purpose of this analysis was technical validation and to estimate odds ratios (ORs). We analysed the dosage data, including sex and the first 20 principal components, with PLINK (version 1.07),^26^ and then combined the results from each site again with a fixed-effect meta-analysis.

Conditional analysis was done with FaSTLMM (version 2.0) on variants in the same regions as those defined for the logistic regression. The purpose of the conditional analysis was to establish whether any other genetic variants in the region were associated with the disease phenotype, independent of the strongest signal from that region. We conditioned on the most significant variants within each of the three regions. Sex was included as a covariate in the conditional analysis. We applied Bonferroni correction to control for multiple testing in the conditional analysis and set the threshold for significance at 5 × 10^−6^ (each 1 megabase region contained approximately 10 000 single nucleotide polymorphisms).

To assess the accuracy of the imputation across regions showing signals satisfying genome-wide significance, we did genotyping in a subset of patients included in the meta-analysis and compared hard genotypes with imputation dosage files. We selected a subset of individuals to represent each of the three broad ethnic origins included in our analysis (ie, European ancestry, African-American, and Asian). Genotyping was done with TaqMan (Life Technologies, Carlsbad, CA, USA) for rs28498976, Sanger sequencing for rs6732655, and Kasper KASP (LGC Genomics, Hoddesdon, Hertfordshire, UK) for rs2947349 (appendix), because differences in sequence context required specific genotyping platforms for each single nucleotide polymorphism.

We did enrichment analysis with the interval-based enrichment analysis tool as integrated in the package INRICH (version 1.0).^27^ Briefly, INRICH takes a set of independent, nominally associated genomic intervals and tests for enrichment of predefined gene sets with permutation. We analysed variants with p values less than 1 × 10^−5^ and defined the interval around index single nucleotide polymorphisms with an *r*^2^ threshold of 0·2. Gene sets as defined by gene ontology pathways were tested for enrichment.

### Role of the funding source

The funders had no role in the study design, data collection, data analysis, data interpretation, or writing of the report. The members of the strategy and analysis committees of the International League Against Epilepsy Consortium on Complex Epilepsies had full access to all data in the study. The strategy committee (appendix) of the Consortium takes final responsibility for the decision to submit for publication.

## Results

40 789 participants, comprising 10 064 people with epilepsy from 12 cohorts and 30 725 controls, were studied. After application of our quality control criteria (appendix), we included a total of 34 853 individuals (8696 with epilepsy and 26 157 controls) in the meta-analysis for all epilepsies (table 1).

Principal component analysis suggested that the cohorts clustered in three broad ethnic origins (European, Asian, and admixed African-American), as expected (appendix). We noted an inflation factor of 1·031, suggesting adequate control for possible cryptic stratification (appendix).

**Figure 1:**
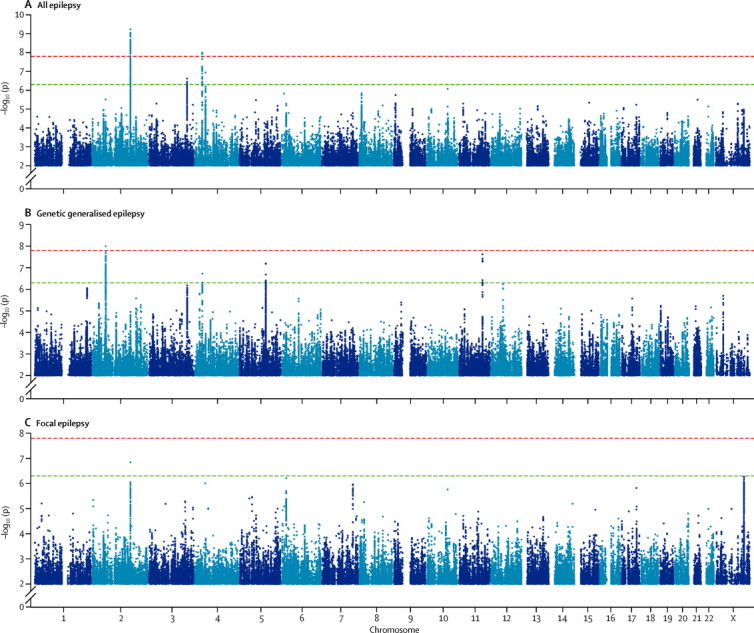
Manhattan plots for meta-analyses of all epilepsy (A), genetic generalised epilepsy (B), and focal epilepsy (C) The red line shows our threshold of significance set at p=1·66  × 10^−8^, and the green line shows the suggestive threshold of p=5 × 10^−7^. Y axis is broken in all graphs.

**Figure 2:**
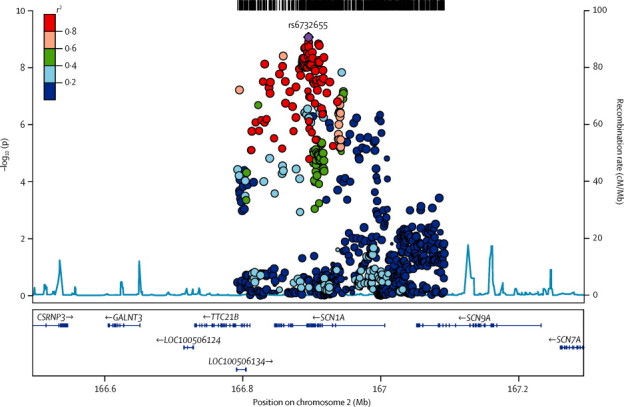
Genomic context of 2q24.3 signal from all-epilepsy analysis Plot created with LocusZoom (version 1.1). Linkage disequilibrium data taken from the 1000 Genomes Project, HG19, March, 2012.

In the all-epilepsy analysis, we identified two loci with genome-wide significance (p<1·66 × 10^−8^; figure 1). The first signal was located at 2q24.3 (figure 2). This signal was centred on the voltage-gated sodium channel gene *SCN1A*, which is a known gene associated with some monogenic epilepsies.^7^, ^28^, ^29^ The most strongly associated variant in this interval was rs6732655 (p=8·71 × 10^−10^, OR 0·89, 95% CI 0·86–0·93; table 2, appendix), located in intron 16 of *SCN1A*. Seventy other variants in this region satisfied the threshold for genome-wide significance. Logistic regression validated the association with 2q24.3 (appendix). The direction of effect was consistent across most cohorts, and there was no evidence of substantial heterogeneity.

**Table 2:** Genome-wide associated loci at p<5·0 × 10^−7^

	**Cytogenetic band**	**Base pair position**	**Allele 1, allele 2**	**Minor allele frequency**	**Candidate gene**	**Annotation**	**Phenotype**	**OR (95% CI)**	**p_LMM_**	**p_cond_**
rs6732655	2q24.3	166895066	T*, A	0·22 (A)	*SCN1A*	Intronic	All epilepsy	0·89 (0·86–0·93)	8·71 × 10^−10^	4·95 × 10^−7^
rs28498976	4p15.1	31151357	A, G*	0·46 (A)	*PCDH7*	Intergenic	All epilepsy	0·90 (0·87–0·94)	5·44 × 10^−9^	2·29 × 10^−4^
rs111577701	3q26.2	167861408	T, C*	0·09 (T)	*GOLIM4*	Intergenic	All epilepsy	1·16 (1·09–1·24)	4·42 × 10^−7^	..
rs535066	4p12	46240287	T, G*	0·40 (G)	*GABRA2*	Intergenic	All epilepsy	1·10 (1·05–1·16)	1·71 × 10^−7^	..
rs2947349	2p16.1	58059803	A*, C	0·26 (C)	*VRK2*/*FANCL*	Intergenic	GGE	1·23 (1·16–1·31)	9·99 × 10^−9^	1 × 10^−4^
rs1939012	11q22.2	102595135	C, T*	0·40 (T)	*MMP8*	Intronic	GGE	1·12 (1·07–1·17)	2·37 × 10^−8^	..
rs1044352	4p15.1	31147874	T*, G	0·50 (T)	*PCDH7*	Synonymous	GGE	0·88 (0·82–0·93)	1·87 × 10^−7^	..
rs55670112	5q22.3	114268470	A, C*	0·47 (C)	None	Intergenic	GGE	1·18 (1·1–1·26)	6·34 × 10^−8^	..
rs12987787	2q24.3	166858391	C, T*	0·21 (C)	*SCN1A*	Intronic	Focal epilepsy	1·12 (1·01–1·14)	1·45 × 10^−7^	..

Base pair position refers to build 37 (hg19). Minor allele frequency is from all poulations from the 1000 Genomes Project. Candidate gene refers to the most plausible candidate gene attributable to the signal. OR corresponds to allele 2, computed from logistic regression. Annotation refers to type of SNP. p_LMM_ refers to p value from linear mixed-model meta-analysis. p_cond_ refers to p value when conditioning on this specific SNP to determine independent signals from same locus. OR=odds ratio. GGE=genetic generalised epilepsy. SNP=single nucleotide polymorphism.

* Ancestral or chimpanzee allele.

In view of the extent of linkage disequilibrium between the variants associated with all epilepsy in the 2q24.3 region (figure 2), we did logistic regression conditioned on the most significant variant identified from the univariate analysis (rs6732655). Our results suggested a tentative independent signal, coming from rs13406236, in an intronic variant in *SCN9A* (p=1·39 × 10^−4^ on conditioning; appendix). We did not identify any further significant signals.

**Figure 3:**
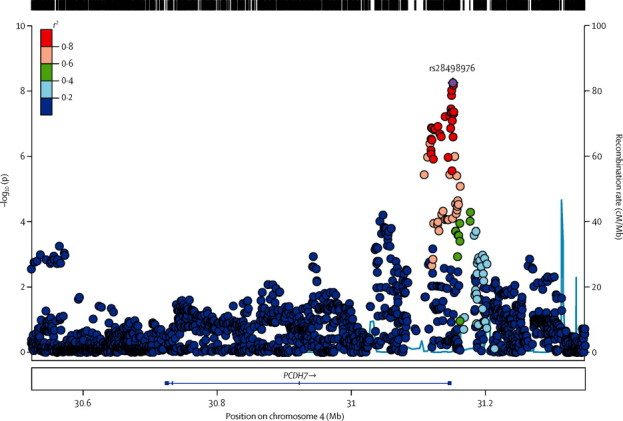
Genomic context of 4p15.1 signal from all-epilepsy analysis Plot created with LocusZoom (version 1.1). Linkage disequilibrium data taken from the 1000 Genomes Project, HG19, March 2012.

A second signal for the all-epilepsy phenotype was located at 4p15.1 and included the 3′ end of the protocadherin gene, *PCDH7* (figure 3). The most strongly associated variant in this region was rs28498976 (p=5·44 × 10^−9^, OR 0·90, 95% CI 0·87–0·94; table 2), located 2·5 kilobases from the 3′ end of *PCDH7*. Logistic regression across *PCDH7* supported the association with this locus (appendix). We noted no additional significant signals from 4p15.1 on conditioning for rs28498976 (appendix). The direction of effect was consistent across all cohorts and we noted no evidence of heterogeneity. Although achieving genome-wide significance only for the all-epilepsy phenotype, the *PCDH7* signal seemed stronger in genetic generalised epilepsy than in focal epilepsy (appendix).

*PCDH7* encodes a calcium-dependent adhesion protein, not previously associated with epilepsy. It is a member of the cadherin gene family. The gene is expressed in the CNS, specifically in thalamocortical circuits and the hippocampus,^30^, ^31^ and expression of *PCDH7* is controlled by *MECP2,*^32^ mutations in which cause Rett syndrome. The cytoplasmic domain of the PCDH7 protein binds to protein phosphatase 1α (PPP1CA), which is enriched in dendritic spines and is important in learning and memory,^33^ and to template activation factor 1 (TAF1), which along with PCDH7 is involved in neurite extension.^34^, ^35^

Suggestive signals of note (p<5 × 10^−7^) for the all-epilepsy phenotype were detected at 3q26.2 (p=4·42 × 10^−7^) and 4p12 (p=1·71 × 10^−7^; table 2). The 3q26.2 region contained the 5′ end of *GOLIM4* (appendix). This gene encodes Golgi internal membrane protein 4, which is degraded when manganese increases above normal concentrations, suggesting a role for this protein in manganese homoeostasis.^36^ Almost all brain manganese is bound to glutamine synthetase, an enzyme playing a key part in production or degradation of the neurotransmitters glutamate, glutamine, and GABA. Decreased brain glutamine synthetase and manganese concentrations have been reported in epilepsy.^37^, ^38^ The 4p12 region contained the 3′ end of the GABA receptor, α2-subunit gene (*GABRA2*). Mutations in other GABA receptors have been reported to cause epilepsy.^39^

After quality control, we included 21 596 individuals (2606 cases and 18 990 controls) across eight cohorts in the meta-analysis of genetic generalised epilepsy (table 1), a subset of those included in the all-epilepsy analysis. Results from the genetic generalised epilepsy meta-analysis suggested an inflation factor of 1·05 (appendix).

**Figure 4:**
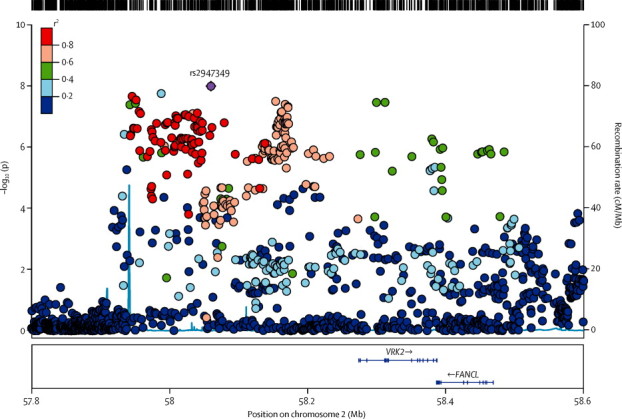
Genomic context of 2p16.1 signal from analysis of genetic generalised epilepsy Plot created with LocusZoom (version 1.1). Linkage disequilibrium data taken from the 1000 Genomes Project, HG19, March 2012.

A single signal achieved the threshold of genome-wide significance (figure 1). Located at 2p16.1, the interval contained genes encoding vaccinia-related kinase 2 (*VRK2*) and Fanconi anaemia, complementation group L (*FANCL*; figure 4). The most strongly associated variant in this region was the intergenic variant rs2947349 (p=9·99 × 10^−9^, OR 1·23, 95% CI 1·16–1·31; table 2). Logistic regression analysis supported the association with 2p16.1 (appendix). We noted no additional significant signals from 2p16.1 on conditioning for rs2947349 (appendix). The direction of effect was consistent across all cohorts, and the association seemed to be specific to genetic generalised epilepsy (appendix).

VRK2 is a serine-threonine protein kinase involved in signal transduction and apoptosis.^40^, ^41^ Variation in *VRK2* has previously been suggested as a risk factor for epilepsy^16^ and schizophrenia.^42^, ^43^, ^44^ Indeed, the schizophrenia-associated risk variant (rs2312147)^43^ shows also a strong signal for genetic generalised epilepsy (p=2·3 × 10^−6^, OR 1·22, 95% CI 1·14–1·30) and is in high linkage disequilibrium with the strongest variant for genetic generalised epilepsy (*r*^2^=0·82), although the direction of the effect is opposite (ie, the protective variant for epilepsy raises risk for schizophrenia). The EPICURE cohort, in which 2p16.1 was originally proposed as a risk factor for genetic generalised epilepsy, was included in our meta-analysis. After exclusion of the EPICURE cohort, the top single nucleotide polymorphism from their study (rs13026414)^16^ remained nominally significant at p=7 × 10^−3^ here. These results provide further support to the suggestion that *VRK2* is a risk locus for both epilepsy and schizophrenia. The other gene in the region, *FANCL*, codes for a RING-type E3 ubiquitin ligase of the Fanconi anaemia pathway. FANCL mono-ubiquitinates FANCD2 and FANCI, proteins involved in DNA repair and homologous recombination.^45^ FANCL has not been previously implicated in epilepsy or any seizure-related phenotype.

We detected suggestive evidence for association with genetic generalised epilepsy at 4p15.1 (p=1·87 × 10^−7^), 5q22.3 (p=6·34 × 10^−8^), and 11q22.2 (p=2·37 × 10^−8^; table 2). The 4p15.1 *PCDH7* signal was the same as that with genome-wide significance for the all-epilepsy phenotype (figure 3, appendix). The 5q22.3 signal was intergenic (appendix). The 11q22.2 signal contained the 5′ end of the matrix metallopeptidase gene *MMP8* (appendix). The direction of effect was consistent across all cohorts and seemed specific to genetic generalised epilepsy (appendix). With a p value of 2·37 × 10^−8^, the 11q22.2 signal reached the conventional threshold for genome-wide significance (p<5 × 10^−8^), but not our more stringent value (p<1·66 × 10^−8^). Matrix metallopeptidases are zinc-dependent endopeptidases involved in the breakdown of the extracellular matrix in physiological processes and in blood–brain inflammation.^46^ Increased expression of MMPs has been recorded in various neurological disease states,^47^ and epileptogenesis is decreased in *MMP9* knockout mice but increased in transgenic rats overexpressing MMP9.^48^

After quality control, we included 28 916 individuals (5310 cases and 23 606 controls) from ten cohorts in our meta-analysis of focal epilepsy. No signal achieved genome-wide significance. Results from the focal meta-analysis suggested an inflation factor of 1·014 (appendix). We observed one notable subthreshold signal (rs12987787, p=1·45 × 10^−7^) from 2q24.3, the region containing *SCN1A* (appendix).

Targeted genotyping of the three GWAS-significant signals supported the accuracy of imputation, with a minimum correlation of 0·98 noted between experimentally determined and imputed genotypes (appendix).

Assessment of enrichment of gene ontology terms for regions containing variants with nominally significant p values (p<1 × 10^−5^) for each of the three phenotypes showed enrichment in several signalling pathways (appendix). Although none of these variants remained significant after correction for multiple testing, our results suggest pathways with biological plausibility.

Finally, we investigated whether any of the four susceptibility loci at nominal genome-wide significance (p<5 × 10^−8^) were associated with outcome of newly treated epilepsy with use of data from Speed and colleagues.^21^ We used both the index single nucleotide polymorphism (table 2) and single nucleotide polymorphisms within a 20 kilobase window around each of the five genes (*SCN1A, PCDH7, VRK2*/*FANCL*, and *MMP8*; appendix). The minimum p value of association with outcome of newly treated epilepsy for any susceptibility locus was 8·14 × 10^−4^ (*MMP8*). We noted no evidence for an association between *SCN1A* (the gene that codes for the target of sodium-channel-blocking class antiepileptic drugs) and epilepsy outcome.

## Discussion

In this genome-wide association meta-analysis of epilepsy and its most common subtypes, we identified three loci with genome-wide significance, and our findings suggest that some loci might be specifically associated with an epilepsy type.

In the whole cohort consisting of all epilepsy, the region of the sodium channel subunit gene *SCN1A* was clearly associated with the disease. This gene is a well-established cause of genetic epilepsy with febrile seizures plus (GEFS+),^28^, ^29^ a generally mild, familial form of epilepsy, and with Dravet syndrome, a severe epileptic encephalopathy usually arising from de-novo mutations.^7^ *SCN1A* was associated with mesial temporal lobe epilepsy and hippocampal sclerosis with febrile seizures in a recent GWAS^14^ and in a meta-analysis of *SCN1A* rs3812718.^49^ *SCN1A* mutations have also been reported in a range of paroxysmal neurological disorders including familial hemiplegic migraine^50^ and, more rarely, in some focal epilepsies.^51^ Whether this robust association with all epilepsy is a true common variant association or a synthetic association due to tagged rare variants in cases with GEFS+ is therefore not clear. Although the cohorts might have included individuals from monogenic GEFS+ families with *SCN1A* mutations of large effect, review of the phenotyping data suggested that inclusion of more than a few such cases was unlikely; moreover, *SCN1A* variants have been reported only in about 10% of large GEFS+ families.^52^

Our all-epilepsy analysis identified a second locus (4p15.1) that satisfied our threshold for genome-wide significance. This locus is newly associated with epilepsy and implicates the gene *PCDH7.* This protocadherin gene is a plausible candidate for common forms of epilepsy, as mutations in another protocadherin gene, *PCDH19,* cause epilepsy and mental retardation in female patients.^53^

For the specific category of genetic generalised epilepsy, we noted the association at 2p16.1 that was previously reported in the EPICURE cohort;^16^ this cohort provided about half of our sample for the meta-analysis of this subtype (table 1). The association maintained nominal significance after removal of EPICURE cases for this locus, where the genes *VRK2* and *FANCL* are within close proximity. With our additional samples, we did not note significance for the 17q21 locus reported by EPICURE investigators for genetic generalised epilepsy (appendix).

For the subcategory of focal epilepsy, we did not note any locus with genome-wide significance, consistent with negative findings from the EPIGEN study of focal epilepsy (samples from which were included in our analysis).^13^ However, a signal at 2q24.3 (containing *SCN1A*) in focal epilepsy approached but did not achieve significance (appendix). This signal in focal epilepsy was in high linkage disequilibrium with that noted for all epilepsy (*r*^2^=0·85). Importantly, the 2q24.3 signal for focal epilepsy that we recorded differed to that reported in a recent study of the narrow focal epilepsy phenotype of mesial temporal lobe epilepsy and hippocampal sclerosis with febrile seizures.^14^ rs7587026 (the previously reported variant) was not significant in our analysis of a broader focal epilepsy phenotype consisting of all focal epilepsies (p=0·01; appendix). We also did not note the association at 1q32.1 (implicating *CAMSAP1L1*) that was previously reported in the Hong Kong cohort,^15^ which was included in our sample (appendix). Most patients in this cohort had focal epilepsy due to known lesions.

Consistent with experience of GWAS in other neuropsychiatric disorders, and common disorders in general, this study reinforces the value of large sample sizes. In the epilepsies, electroclinical and imaging data allow the identification of clinical syndromes that share common clinical features. Our study findings suggest that an experimental design that includes fractionation of samples into clinical subtypes can reveal syndrome-specific risk alleles, but the identification of these alleles will be assisted by the collection and genotyping of larger sample sizes. Although this lumping versus splitting debate in genetic analyses is not unique to the epilepsies, there has been long-standing controversy about it in clinical epileptology,^54^ which genetics will help to inform.

Limitations of our study include sample size; although ours is large, even larger samples have yielded more findings in other disorders.^55^, ^56^, ^57^ Larger samples would enable further analysis of epilepsy subtypes, and the International League Against Epilepsy Consortium on Complex Epilepsies now provides a useful vehicle for future efforts. Second, our meta-analysis relied on genotypes generated separately on various platforms, an issue common to most meta-analyses. Third, extension of the phenotyping data to include treatment outcome would be ideal, but in a cross-sectional cohort this approach has methodological difficulties. Finally, we did not have an independent replication sample. However, stringent criteria for statistical significance were set a priori, and for loci achieving our threshold of genome-wide significance the direction of effects were largely consistent across the cohorts, and extended over multiple variants in high linkage disequilibrium.

Taken together, these data show that, with sufficient sample size, susceptibility loci for common epilepsies can be identified through the analysis of common variation. The role of rare variants of large effect is also well established, particularly in rarer Mendelian epilepsies.^3^, ^4^, ^5^, ^6^, ^7^ The role of rare variants in the common epilepsies is at present under exploration by deep-sequencing approaches.^11^, ^58^, ^59^ A dual approach of identification of both rare and common variation will result in improved understanding of the genetic architecture for the overall population of people with epilepsy, necessary for precision medicine. Although our findings will not be of immediate clinical usefulness, they are an important first step to understand the genetic architecture of the epilepsies, which could lead to clinically relevant markers of prognosis and outcome.
