# Supplementary material for: Genetic determinants of common epilepsies: a meta-analysis of genome-wide association studies
Source: Lancet Neurol. 2014 Sep 1;13(9):893–903. doi: 10.1016/S1474-4422(14)70171-1 (PMC4189926; doi:10.1016/S1474-4422(14)70171-1)
Supplement: Supplementary appendix [file mmc1.pdf]

THE LANCET Neurology

Supplementary webappendix

This webappendix formed part of the original submission and has been peer reviewed. We post it as supplied by the authors.

Supplement to: International League Against Epilepsy Consortium on Complex Epilepsies. Genetic determinants of common epilepsies: a meta-analysis of genome-wide association studies. *Lancet Neurol* 2014; published online July 31. http://dx.doi.org/10.1016/S1474-4422(14)70171-1.

Appendix Table of Contents

AUTHOR AFFILIATIONS **2**

SUPPLEMENTARY METHODS **5**

Epilepsy cohorts 5

Control cohorts 7

Imputation 7

Principal components analysis 7

Association 7

Meta-analysis 8

Power Calculations 8

Conditional analysis 8

Logistic regression 8

Enrichment analysis 8

SUPPLEMENTARY FIGURES **9**

Supplementary Figure 1: Principal components analysis of cases and controls. 9

Supplementary Figure 2: Power calculations. 10

Supplementary Figure 3: Q-Q plots for meta analysis. 11

Supplementary Figure 4: Magnitude and direction of rs6732655 (*SCN1A*). 13

Supplementary Figure 5. Logistic regression. Gender and first 20 PCAs included as covariates 14

Supplementary Figure 6. Conditional analysis. Gender included as co-variate. 15

Supplementary Figure 7: Magnitude and direction of rs28498976 (*PCDH7*) 16

Supplementary Figure 8: Sub-threshold signal at 3q26.2 (all epilepsy). 17

Supplementary Figure 9: Sub-threshold signal at 4p12 (all epilepsy) 17

Supplementary Figure 10: Magnitude and direction of rs2947349 (*VRK2*) 18

Supplementary Figure 11: Sub-threshold signal at 4p15.1 (GGE). 19

Supplementary Figure 12: Sub-threshold signal at 5q22.3 (GGE). 19

Supplementary Figure 13: Sub-threshold signal at 11q22.2 (GGE). 20

Supplementary Figure 14: Magnitude and direction of rs1939012 (*MMP8*) 21

Supplementary Figure 15: Sub-threshold signal at 2q24.3 (focal). 22

Supplementary Figure 16: Magnitude and direction of rs72823592 (17q21). 23

Supplementary Figure 17: Magnitude and direction of rs7587026 (*SCN1A*). 24

Supplementary Figure 18: Magnitude and direction of rs2292096 (*CAMSAP1L1*). 25

SUPPLEMENTARY TABLES **26**

Supplementary Table 1: Details of pre-quality control numbers 26

Supplementary Table 2: Control cohorts. 27

Supplementary Table 3: Confirmatory genotyping. 27

Supplementary Table 4 A/B/C: Results of enrichment analysis 28

Supplementary Table 5: Susceptibility loci (p<5x10-8) with outcome of newly treated epilepsy.33

SUPPLEMENTARY REFERENCES **34**

AUTHOR AFFILIATIONS

The International League Against Epilepsy Consortium on Complex Epilepsies
Richard J L Anney[1], Andreja Avbersek[2], David Balding[3], Larry Baum[4], Felicitas Becker[5], Samuel F Berkovic[6], Jonathan P Bradfield[7], Lawrence C Brody[8], Russell J Buono[7,9,10], Claudia B Catarino[2], Gianpiero L Cavalleri[11], Stacey S Cherny[12], Krishna Chinthapalli[2], Alison J Coffey[13], Alastair Compston[14], Patrick Cossette[15], Gerrit-Jan de Haan[16], Peter De Jonghe[17], Carolien G F de Kovel[18], Norman Delanty[11,19], Chantal Depondt[20], Dennis J Dlugos[21], Colin P Doherty[22], Christian E Elger[23], Thomas N Ferraro[9,24], Martha Feucht[25], Andre Franke[26], Jacqueline French[27], Verena Gaus[28], David B Goldstein[29,30], Hongsheng Gui[12], Youling Guo[12], Hakon Hakonarson[7,31], Kerstin Hallmann[23,32], Erin L Heinzen[29,33], Ingo Helbig[34], Helle Hjalgrim[35], Margaret Jackson[36], Jennifer Jamnadas-Khoda[2], Dieter Janz[28], Michael R Johnson[37], Reetta Kälviäinen[38,39], Anne-Mari Kantanen[40], Dalia Kasperavičiūte[2,41], Dorothee Kasteleijn-Nolst Trenite[18], Bobby P C Koeleman[18], Wolfram S Kunz[23], Patrick Kwan[42,43], Yu Lung Lau[44], Anna-Elina Lehesjoki[45], Holger Lerche[5], Costin Leu[2], Wolfgang Lieb[46], Dick Lindhout[16,18], Warren Lo[47], Daniel H Lowenstein[48], Alberto Malovini[49], Anthony G Marson[50], Mark McCormack[11], James L Mills[51], Martina Moerzinger[25], Rikke S Møller[34,52], Anne M Molloy[53], Hiltrud Muhle[34], Mark Newton[54], Ping-Wing Ng[55], Markus M Nöthen[56], Peter Nürnberg[57], Terence J O'Brien[42], Karen L Oliver[6], Aarno Palotie[58], Faith Pangilinan[8], Katharina Pernhorst[59], Slave Petrovski[29,42], Michael Privitera[60], Rodney Radtke[61], Philipp S Reif[62], Felix Rosenow[62], Ann-Kathrin Ruppert[57], Thomas Sander[28,57], Theresa Scattergood[63], Steven Schachter[64], Christoph Schankin[65], Ingrid E Scheffer[6,66], Bettina Schmitz[28], Susanne Schoch[59], Pak C Sham[12], Sanjay Sisodiya[2,67], David F Smith[68], Philip E Smith[69], Doug Speed[3], Michael R Sperling[70], Michael Steffens[71], Ulrich Stephani[34], Pasquale Striano[72], Hans Stroink[16], Rainer Surges[23], K Meng Tan[42], The KORA study group[73], G Neil Thomas[74], Marian Todaro[42], Anna Tostevin[2], Rossana Tozzi[75], Holger Trucks[57], Frank Visscher[76], Sarah von Spiczak[34], Nicole M Walley[29], Yvonne G Weber[5], Zhi Wei[77], Christopher Whelan[11], Wanling Yang[44], Federico Zara[78], Fritz Zimprich[79]

1. Department of Psychiatry, Trinity College Dublin, Trinity Centre for Health Sciences, St. James's Hospital, Dublin 8, Ireland.
2. Department of Clinical and Experimental Epilepsy, UCL Institute of Neurology, Queen Square, London WC1N 3BG, UK.
3. UCL Genetics Institute, University College London WC1E 6BT, UK.
4. School of Pharmacy, The Chinese University of Hong Kong, Shatin, Hong Kong.
5. Department of Neurology and Epileptology, Hertie Institute for Clinical Brain Research, University of Tübingen, Tübingen, Germany.
6. Epilepsy Research Centre, University of Melbourne, Austin Health, Heidelberg, Australia.
7. Center for Applied Genomics, The Children's Hospital of Philadelphia, Philadelphia, PA, USA.
8. Genome Technology Branch, National Human Genome Research Institute, National Institutes of Health, Bethesda, Maryland, United States of America.
9. Department of Biomedical Sciences, Cooper Medical School of Rowan University Camden, NJ, USA.
10. Department of Neurology Thomas Jefferson University Hospital Philadelphia, PA, USA.
11. Molecular and Cellular Therapeutics, The Royal College of Surgeons in Ireland, Dublin, Ireland.
12. Department of Psychiatry, The University of Hong Kong, Hong Kong.
13. The Wellcome Trust Sanger Institute, Hinxton, Cambridge, UK.
14. Department of Clinical Neurosciences, University Neurology Unit, Addenbrooke's Hospital, Cambridge, UK.
15. Department of Neurosciences, University of Montreal, Montreal QC, Canada.
16. SEIN Epilepsy Institute in The Netherlands, Hoofddorp, The Netherlands.
17. Neurogenetics Group, Department of Molecular Genetics, VIB and Laboratory of Neurogenetics, Institute Born-Bunge, University of Antwerp, Antwerp, Belgium.
18. Department of Medical Genetics, University Medical Center Utrecht, Utrecht, The Netherlands.
19. The Division of Neurology, Beaumont Hospital, Dublin, Ireland.
20. Department of Neurology, Hôpital Erasme, Université Libre de Bruxelles, 808 Route de Lennik, 1070 Brussels, Belgium.
21. Division of Child Neurology, The Children's Hospital of Philadelphia, Departments of Neurology and Pediatrics, Perelman School of Medicine at the University of Pennsylvania, Philadelphia, PA, USA.
22. Neurology Department, St. James's Hospital, Dublin, Ireland.

23. Department of Epileptology, University of Bonn, Bonn, Germany.
24. Dept of Pharmacology and Dept of Psychiatry, University of Pennsylvania Perlman School of Medicine, Philadelphia, PA, USA.
25. Department of Pediatrics, Medical University of Vienna, Vienna, Austria.
26. Institute of Clinical Molecular Biology Christian-Albrechts-University of Kiel, University Hospital Schleswig Holstein, Kiel, Germany.
27. Department of Neurology, NYU School of Medicine, New York City, USA.
28. Department of Neurology, Charité Universitaetsmedizin Berlin, Campus Virchow-Clinic, Berlin, Germany.
29. Center for Human Genome Variation, Duke University School of Medicine, Durham, North Carolina, USA.
30. Department of Molecular Genetics and Microbiology, Duke University School of Medicine, Durham, North Carolina, USA.
31. Department of Pediatrics, The Perelman School of Medicine, University of Pennsylvania, Philadelphia, PA, USA.
32. Department of Genomics, Life & Brain Center, University of Bonn Medical Center, Bonn, Germany.
33. Department of Medicine, Section of Medical Genetics, Duke University, School of Medicine, Durham, North Carolina, USA.
34. Department of Neuropediatrics, University Medical Center Schleswig-Holstein (UKSH), Kiel, Germany.
35. Institute of Regional Health Services Research, University of Southern Denmark, Odense, Denmark and Danish Epilepsy Centre, Dianalund, Denmark.
36. The Royal Victoria Infirmary, Newcastle Upon Tyne Hospitals NHS Trust, UK.
37. Division of Brain Science, Imperial College London, London, UK.
38. Department of Neurology, School of Medicine, University of Eastern Finland.
39. Kuopio Epilepsy Center, Kuopio University Hospital, Kuopio, Finland.
40. Neurocenter, Kuopio University Hospital, Kuopio, Finland.
41. Institute of Clinical Sciences, Imperial College London, London, UK.
42. The Melbourne Brain Centre, The Department of Medicine, The Royal Melbourne Hospital, The University of Melbourne, Victoria, Australia.
43. Department of Medicine and Therapeutics, The Chinese University of Hong Kong, Shatin, Hong Kong.
44. Department of Paediatrics and Adolescent Medicine, The University of Hong Kong, Hong Kong.
45. Neuroscience Center and Research Program for Molecular Neurology, University of Helsinki and Folkhälsan Institute of Genetics, Helsinki, Finland.
46. Institut für Epidemiologie, Christian-Albrechts-Universität zu Kiel, Kiel, Germany.
47. Department of Pediatrics and Neurology, The Ohio State University and Nationwide Children's Hospital, Columbus Ohio, USA.
48. Department of Neurology, University of California, San Francisco, San Francisco, California, USA.
49. Department of Industrial and Information Engineering, University of Pavia, Pavia, Italy and IRCCS Fondazione Salvatore Maugeri, Pavia, Italy.
50. Department of Molecular and Clinical Pharmacology, University of Liverpool, UK.
51. Division of Epidemiology, Statistics, and Prevention Research, Eunice Kennedy Shriver National Institute of Child Health and Human Development, National Institutes of Health, Bethesda, Maryland, USA.
52. Wilhelm Johannsen Centre for Functional Genome Research, Copenhagen, Denmark.
53. School of Medicine, Trinity College, Dublin, Ireland.
54. Department of Neurology, Austin Health, Victoria, Australia.
55. United Christian Hospital, Hong Kong.
56. Institute of Human Genetics, University of Bonn Medical Center, Bonn, Germany.
57. Cologne Center for Genomics, University of Cologne, Cologne, Germany.
58. Institute for Molecular Medicine Finland (FIMM), University of Helsinki, Finland and The Broad Institute of MIT and Harvard, Cambridge, USA.
59. Department of Neuropathology, University of Bonn Medical Center, Bonn, Germany.
60. Department of Neurology, Neuroscience Institute, University of Cincinnati Medical Center, Cincinnati, OH, USA.
61. Department of Medicine, Division of Neurology, Duke University School of Medicine, Durham, North Carolina, USA.

62. Epilepsy-Centre Hessen, Department of Neurology, University Medical Center Giessen and Marburg, Marburg, Germany and Philipps-University Marburg, Marburg, Germany.
63. Department of Endocrinology, Hospital of The University of Pennsylvania, Philadelphia, PA, USA.
64. Departments of Neurology, Beth Israel Deaconess Medical Center, Massachusetts General Hospital, and Harvard Medical School, Boston, MA, USA.
65. Department of Neurology, University of Munich Hospital – Großhadern, Munich, Germany.
66. Florey Institute and Department of Pediatrics, Royal Children's Hospital, University of Melbourne, Victoria, Australia.
67. Epilepsy Society, Chalfont-St-Peter, Bucks, UK
68. The Walton Centre NHS Foundation Trust, Liverpool, UK.
69. Department of Neurology, Alan Richens Epilepsy Unit, University Hospital of Wales, Cardiff, UK.
70. Department of Neurology and Comprehensive Epilepsy Center, Thomas Jefferson University, Philadelphia, PA, USA.
71. Research Division, Federal Institute for Drugs and Medical Devices (BfArM), Bonn, Germany.
72. Pediatric Neurology and Muscular Diseases Unit, Department of Neurosciences, Rehabilitation, Ophthalmology, Genetics, Maternal, and Child Health, G. Gaslini Institute, University of Genoa, Genoa, Italy.
73. Helmholtz Institute, Munchen, Germany. The KORA-Study Group consists of A. Peters (speaker), J. Heinrich, R. Holle, R. Leidl, C. Meisinger, K. Strauch, and their co-workers C. Gieger and H. Grallert, who are responsible for the design and conduct of the KORA studies.
74. School of Health and Population Sciences, College of Medical and Dental Sciences, The University of Birmingham, Birmingham, UK.
75. C. Mondino National Neurological Institute, Pavia, Italy.
76. Department of Neurology, Admiraal De Ruyter Hospital, Goes, The Netherlands.
77. Department of Computer Science, New Jersey Institute of Technology, New Jersey, USA.
78. Department of Neurosciences and Rehabilitation, G. Gaslini Institute, Genoa, Italy.
79. Department of Neurology, Medical University of Vienna, Vienna, Austria.

SUPPLEMENTARY METHODS

Epilepsy cohorts

Epilepsy cohorts contributing to the meta-analysis are detailed below.

EPIGEN (Reported by – Chantal Depondt, Sanjay Sisodiya, Norman Delanty, Gianpiero Cavalleri, Erin Heinzen and David Goldstein)
The EPIGEN study consisted of epilepsy cohorts from Beaumont Hospital Dublin (Ireland), Université Libre de Bruxelles (ULB, Belgium), Duke University Medical Centre (North Carolina, USA) and University College Hospital London (UK).

Inclusion Criteria: Except for Duke, only adult (>16 years) patients with epilepsy were recruited.
Exclusion Criteria: No specific exclusion criteria.
Quality assurance: At all sites, subjects were recruited and phenotyped by experienced epilepsy specialists. At Duke, all cases underwent independent case-record review by an epilepsy nurse specialist, and ambiguous diagnoses were re-evaluated by a second epileptologist. If the diagnosis remained unclear, then the patient was excluded from the study. For London, all cases underwent review by independent epileptologists. For Brussels, study PI (Chantal Depondt) reviewed the classification of all cases by case-note review. For Dublin, no systematic quality assurance was undertaken.

Site-specific details for each EPIGEN cohort as organized for the analysis are as follows:

- **EPIGEN-Dublin**

Patients were recruited from a specialized epilepsy clinic at Beaumont Hospital, Dublin, Ireland. Patients were mostly of Irish ethnicity. Patients were genotyped on the Illumina platform using a combination of chips (610-Quad+550+300v1/Omni1-Quad).

- **EPIGEN-Brussels**

Patients were recruited from epilepsy clinics at UZ Gasthuisberg, Katholieke Universiteit Leuven, and Hôpital Erasme, Université Libre de Bruxelles. Patients were largely of Belgian ethnicity. Patients were genotyped on the Illumina platform using a combination of chips (610-Quad/300 V1 & V2).

- **EPIGEN-Duke**

Patients were recruited from outpatient clinics at Duke University Medical Center, Durham, North Carolina. Clinical assessment was by standardized patient interview. Patients were mostly of European Caucasian or African American ancestry. Patients were genotyped on the Illumina platform using a combination of chips (610-Quad/550+300+610+iselect-Quad).

- **EPIGEN-London**

Patients were recruited from outpatient specialist epilepsy clinics at the National Hospital for Neurology and Neurosurgery. Patients were genotyped on the Illumina platform using a combination of chips (610-Quad, 550/1.2M).

EPICURE (Reported by - Pasquale Striano, Federico Zara, Thomas Sander and Wolfram Kunz)
Inclusion criteria: Epilepsy patients aged $\geq$3 years of European ancestry with common GGE syndromes (CAE, JAE, JME, and EGTCS alone with documented GSW-EEG) were recruited as a concerted effort of national and international epilepsy genetics programs integrated in the European EPICURE Project as previously described[1]. EPICURE Partners are listed at; http://www.epicureproject.eu/html/partners/default.aspx.
Exclusion criteria: Individuals with a history of severe major psychiatric disorders, severe intellectual disability or structural, metabolic or degenerative brain disorders.
Quality Assurance: Phenotyping and diagnostic classification of GGE syndromes were carried out by epilepsy specialists according to standardized protocols available at; http://portal.ccg.uni-koeln.de/ccg/research/epilepsy-genetics/sampling-procedure. All subjects were phenotyped by experienced epilepsy specialists.

Patients were genotyped using the Affymetrix 6.0 platform.

Hong Kong Cohort (Reported by - Patrick Kwan, Stacey Cherny and Larry Baum)
Inclusion criteria: Epilepsy patients of Han Chinese ancestry aged $\geq$2 years were recruited by epilepsy specialists in five regional hospitals in Hong Kong.
Exclusion criteria: Significant psychiatric comorbidity, psychogenic non-epileptic seizures, alcohol or illicit drug misuse.
Quality assurance: All subjects were recruited by epilepsy specialists and study PI (Patrick Kwan) reviewed the classification of all cases by case-note review.

Patients were genotyped on the Illumina platform using Illumina 610 chip.

Philadelphia Cohort (Reported by – Mike Sperling, Dennis Dlugos, Warren Lo, Russell Buono and Hakon Hakonarson)

Inclusion criteria: Patients with GGE or non-symptomatic focal epilepsy aged $\geq$3 years were recruited in two previous studies. (A) Genetic Influences on Human Epilepsy (GIHE) 2001-2006 collected 1971 total samples with 951 from patients and 1020 from first degree relatives or unrelated controls. Seven clinical sites were involved: Thomas Jefferson University Hospital, Philadelphia PA; The Children's Hospital of Philadelphia (CHOP); Nationwide Children's Hospital, Columbus Ohio; The Hospital of the University of Pennsylvania, Philadelphia, Pennsylvania; University of Cincinnati, Cincinnati Ohio; University of Montreal, Montreal Quebec Canada; and Beth Israel Deaconess/Harvard University, Boston, Massachusetts. (B) Genetic Study of Common Forms of Epilepsy (GSCFE) 2009-2012 collected 1013 additional patient samples from the top three clinical collection sites in GIHE: Thomas Jefferson University Hospital, The Children's Hospital of Philadelphia (CHOP) and Nationwide Children's Hospital. Clinical inclusion criteria were published previously[2]. Patients were of mostly European Caucasian and African American ancestry.
Exclusion Criteria: Patients with symptomatic focal epilepsy.
Quality Assurance: All patients were recruited by epilepsy specialists, according to a standardized protocol.

Note that the broad 'Philadelphia' cohort was divided into four subcohorts post quality control, based on ethnicity and genotyping platform (see Table 1 in main text). They are:

- Philadelphia_550_AA – individuals of African-American ancestry, genotyped on Illumina 550 platform.
- Philadelphia_550_CAU – individuals of European-American ancestry, genotyped on Illumina 550 platform.
- Philadelphia_Omni_AA - individuals of African-American ancestry, genotyped on Illumina Omni platform.
- Philadelphia_Omni_CAU – individuals of European-American ancestry, genotyped on Illumina Omni platform.

Healthy controls were recruited by the Center for Applied Genomcis at CHOP. A total of 6,419 European Caucasian and 2,844 African American subjects were recruited and genotyped and used as controls in the case-control analysis performed. Both patients and controls were genotyped on the Illumina platform using a combination of Illumina chips (550, 610-quad and Omni-Express), with platform matching performed between cases and controls prior to analysis.

Imperial – Liverpool – Melbourne (ILM) Collaboration (Reported by Michael Johnson, Terence O'Brien, Anthony Marson, Slave Petrovski and Aarno Palotie)
Inclusion criteria: Epilepsy patients were recruited to UK and Australian prospective cohorts of newly treated epilepsy as previously described[3], and to a pharmacogenetic study of patients who had taken clobazam or vigabatrin or were starting clobazam prospectively (CLOPS/VIPS). Patients were mostly of European Caucasian ancestry.
Exclusion criteria: Epilepsy patients with progressive structural brain lesions.
Quality assurance: UK and Australian epilepsy cases as previously described[3]. For CLOPS/VIPS, all patients were recruited by epilepsy specialists following case-note review by an epileptologist according to a standardized protocol.

Patients were genotyped using Illumina 660-quad.

GenEPA (Reported by - Reeta Kälviäinen)
Inclusion Criteria: Patients of Finish ancestry with a confirmed diagnosis of temporal lobe epilepsy (TLE).
Exclusion criteria: Patients with progressive structural lesions.
Quality assurance: All patients were recruited from a single regional epilepsy centre by experienced epilepsy specialists. All clinical data including MRI and EEG results uploaded to a central research database and reviewed by an independent epilepsy specialist.

Patients were of Finnish ancestry and genotyped on the Illumina 610-quad.

Control cohorts

Control samples for this project consisted of the cohorts described in Supplementary Table 2 below.

Imputation

Imputation was conducted at individual analytical sites using a pre-agreed, standardized protocol. Before imputation, we filtered GWAS data based on the following quality control metrics: Hardy Weinberg Equilibrium ($<1x10^{-6}$), sample missingness rate (<95%), minor allele frequency (<1%), heterozygosity, pairwise relatedness, and gender checks (against expected gender from clinical phenotype). SNP datasets were then converted to NCBI Genome Build 37 (hg19) and the forward strand of the reference genome.

Pre-phasing and imputation was conducted using IMPUTE2[4]. For pre-phasing, we divided populations according to ethnicity. Within a population we then divided chromosomes in to adjacent regions of 5Mb. Where a region had less than 200 SNPs or if a region spanned the centromere, we adjusted accordingly and merged with an adjacent region.

We imputed to the 1000 Genomes Phase I (interim) June 2011 reference. Impute2 offers two choices for imputation: imputing from 'best-guess haplotypes' or imputing from 'a sample of alternate haplotype configurations'. We imputed using 'best-guess haplotypes'.

Principal components analysis

We selected a panel of approximately 3000 SNPs for PCA analysis. SNPs were selected from HapMap by pruning the full complement of 1.8 million variants for r^2 <0.005. Dosage files for these SNPs were extracted from each case cohort and used to calculate principal components.

Association

We chose to employ a linear mixed model (LMM) approach for our analysis[5], as implemented in the software FaST LMM (version 1.09). The key advantage of this method is that it deals with the issues of cryptic relatedness by integrating an inter-individual relatedness matrix into the regression model that accounts for the effects both population structure and residual relatedness in the cohort.

Each analytical site conducted the LMM association analysis following a pre-agreed protocol, on relevant case/control cohorts in Table 1. The protocol detailed four distinct steps; 1) selection of SNPs for calculating relatedness, 2) identify sets of homogenous individuals for inclusion in LMM, 3) preparation of the dosage files for analysis, and 4) running the FaST LMM algorithm.

Step 1: SNPs for calculation of relatedness matrix were selected using PLINK from hard-genotyped GWAS variants from merged case/control cohorts (see Table 1) with the following criteria (across chromosomes 1-22): call rate >99.5%, HWE p-value >0.01, MAF >0.01. From this subset of variants we then pruned (using PLINK command --indep-pairwise 100 25 0.05) for those in low linkage disequilibrium (r^2) using following parameters; 100kb sliding window of 25 SNP step size and an r^2 threshold of 0.05. This generated sets of between 50,000 – 100,000 SNPs.

Step 2: Using the sets of variants identified in Step 1, genetically homogenous individuals for inclusion in the LMM were identified by calculating relationship matrices and plotting using principal components analysis (PCA). Based on the top two PCs, we identified and removed any samples that appeared as outliers in the plots.

Step 3: Dosage files for LMM were prepared by first removing variants with MAF <0.5%, callrate <0.9 and info scores (from IMPUTE2 output) <0.5. We then merged dosage files across case/control cohorts.

Step 4. The LMM analysis was conducted across each phenotype, incorporating gender as a covariant.

Meta-analysis

Fixed effects meta-analysis was conducted using the software package METAL[6]. METAL selects a reference allele for each marker (all studies were aligned to the same reference allele) and calculates (from p values) a z-statistic, which summarizes the magnitude and the direction of effect relative to the reference allele. An overall z-statistic and p-value are then calculated from a weighted sum (proportional to the square-root of the number of individuals examined in each sample) of the individual statistics[6]. Genomic control correction was applied to individual cohorts within METAL. SNPs showing significant amounts of heterogeneity ($p<0.05$) were removed before applying the fixed-effects analysis.

The QQ plots generated from meta-analysis results are show in Supplementary Figures 3a-3c. We observe very strong LD around some of the 9 loci shown in Table 2, with many SNPs in those regions showing elevated significance. After removing SNPs within 2MB of any of those loci (in total representing about 1% of the genome), the QQ plots show little indication of inflation (see Supplementary Figures 3d-3f).

Power Calculations

If a variant explains a proportion r^2 of phenotypic variance (cases 1, controls 0), the test statistic T from linear regression will have a chi-squared distribution with 1 degree of freedom and non-centrality parameter $nr^2/(1-r^2)$, where n is the total number of samples. We declare a variant genome-wide significant if its probability under the null distribution (which assumes T is distributed with non-centrality parameter 0) is less than 1.7e-8; i.e., if $T > 31.8$. Therefore, for different r^2 and n, we can compute the detection power as the probability that $T>31.8$. For Supplementary Figure 2, we convert r^2 to estimates of variance explained on the underlying liability scale[7], taking account the ascertainment present in our GWAS.

Conditional analysis

FastLMM was also used for the conditional regression, using the same steps, except that in addition to gender, the genotypes for the conditioned SNP (dosage values between 0 and 2) were also added as a covariate.

Logistic regression

To verify the FastLMM results, we also performed logistic regression using PLINK on the dosage files. We included sex and 20 PCA as covariates. Logistic regression was run on each of the twelve case/control cohorts, and results meta-analysed using a fixed-effects model. The results from the logistic regression meta-analysis were consistent with those of FastLMM; however, we preferred the latter as it more elegantly takes into account (subtle) population structure and relatedness within the datasets.

Enrichment analysis

Enrichment analysis was conducted using the package INRICH.[8] We considered variants with a p value $< 1 \times 10^{-5}$ and defined intervals around index SNPs using secondary p value < 0.05 and an r^2 threshold of 0.2. Gene sets as defined by GO ontology pathways were tested for enrichment. The INRICH procedure follows three stages; firstly, determine independent, nominally associated genomic intervals using the --clump routine in PLINK. Secondly, INRICH calculates the empirical significance of the observed gene enrichment (EMPIRICAL_P) using an interval-based permutation routine (n = 5000); finally a second permutation is applied to calculate the significance corrected for multiple comparisons (CORRECTED_P) at the gene-set level (N = 2000). Gene-sets were compiled using the Gene Ontology (GO) database. A total of 13610 RefGene gene identifiers were mapped recursively to 5321 GO Terms or gene-sets.

SUPPLEMENTARY FIGURES

Supplementary Figure 1: Principal components analysis of cases and controls considered for epilepsy meta-analysis.

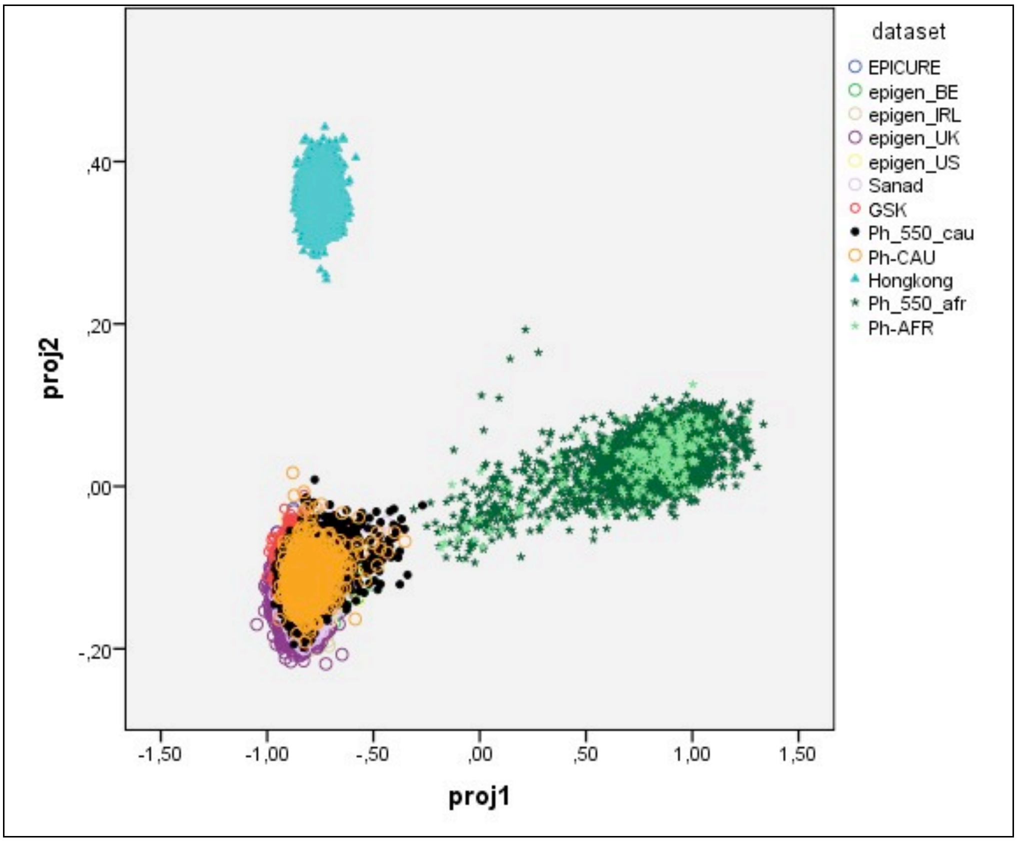

Supplementary Figure 2: Power calculations.

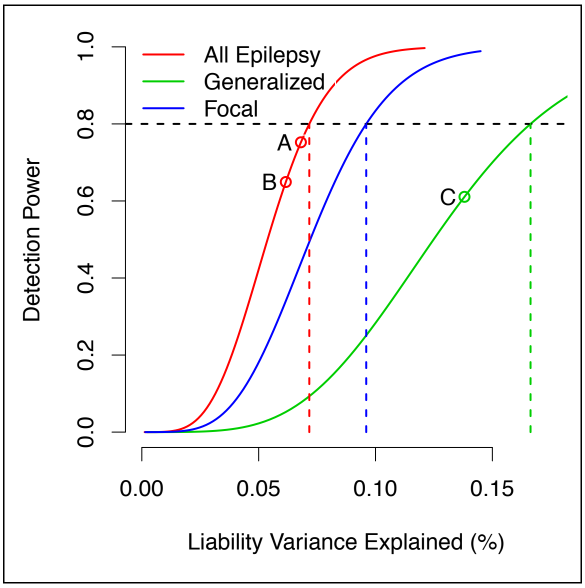

Legend: Dots indicate the liability variance explained by each of the three genome-wide significant signals detected through the meta analysis. A = rs6732655, B = rs28498976, C = rs2947349

Supplementary Figure 3: Q-Q plots for meta analysis.

Supplementary Figure 3a: All epilepsy. Inflation=1.031

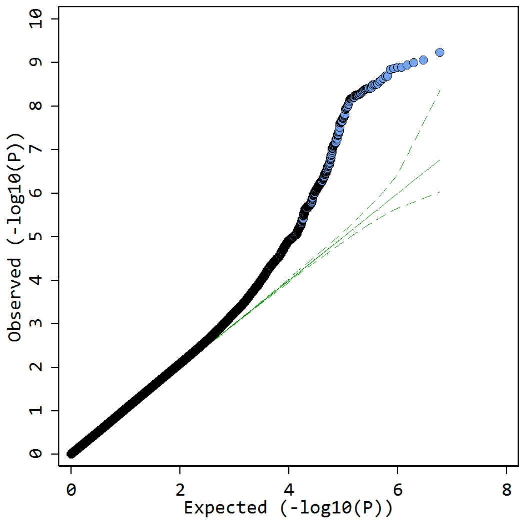

Supplementary Figure 3d: All epilepsy, with top 9 loci removed. Inflation=1.03

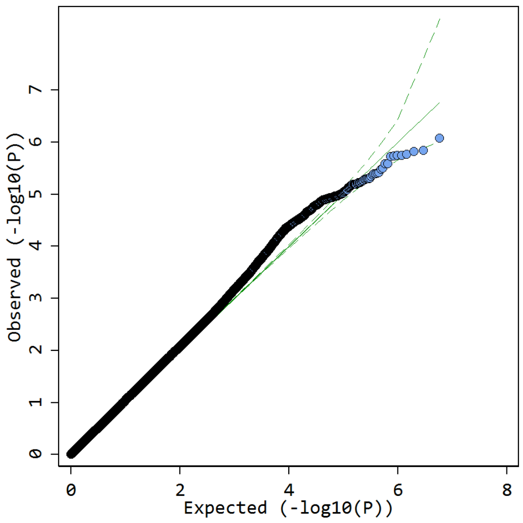

Supplementary Figure 3b: GGE. Inflation=1.050

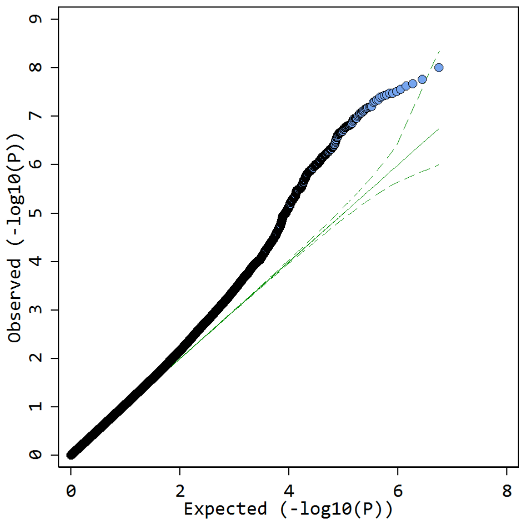

Supplementary Figure 3e: GGE, with top 9 loci removed. Inflation=1.05

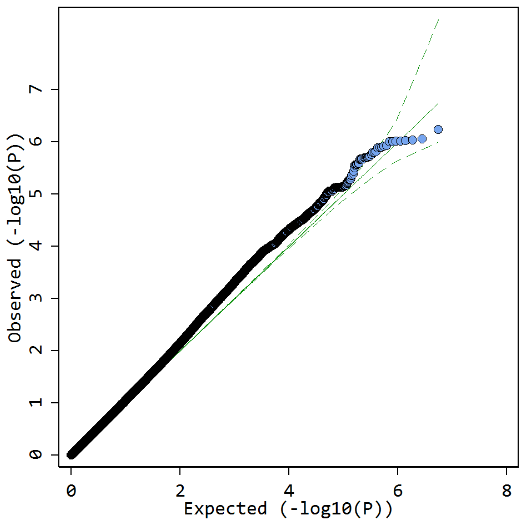

**Supplementary Figure 3c: Focal. Inflation=1.014**	**Supplementary Figure 3f: Focal, with top 9 loci removed. Inflation=1.02**
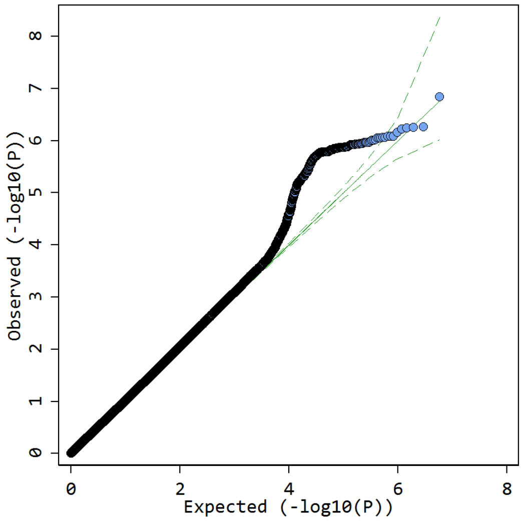	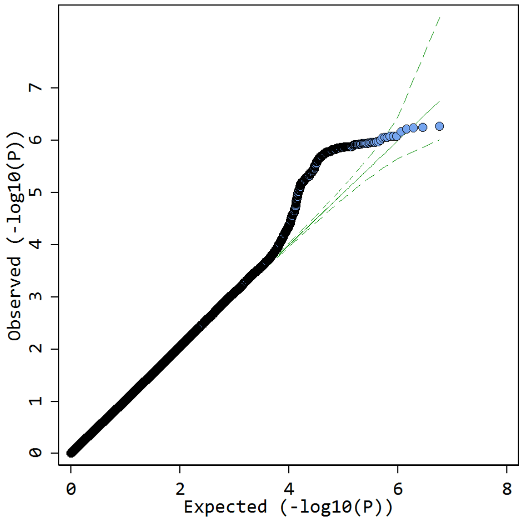

Supplementary Figure 4: Magnitude and direction of rs6732655 (*SCN1A*) across 3 phenotypes.

rs6732655 – All epilepsy – meta p = $8.71x10^{-10}$

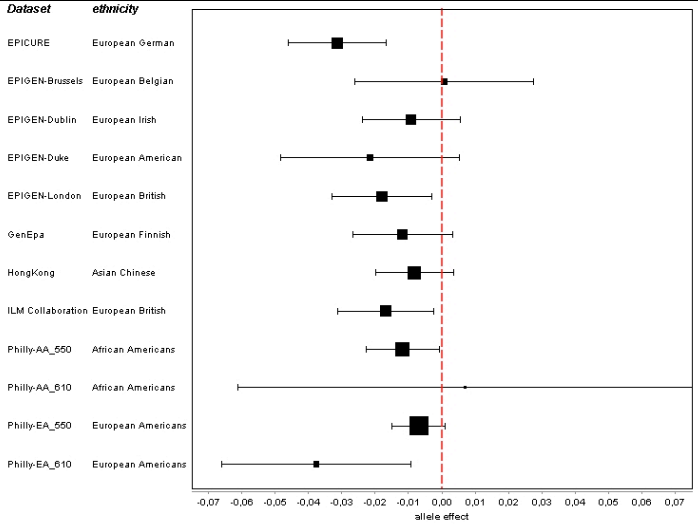

rs6732655 – GGE – meta p = $5.78x10^{-4}$

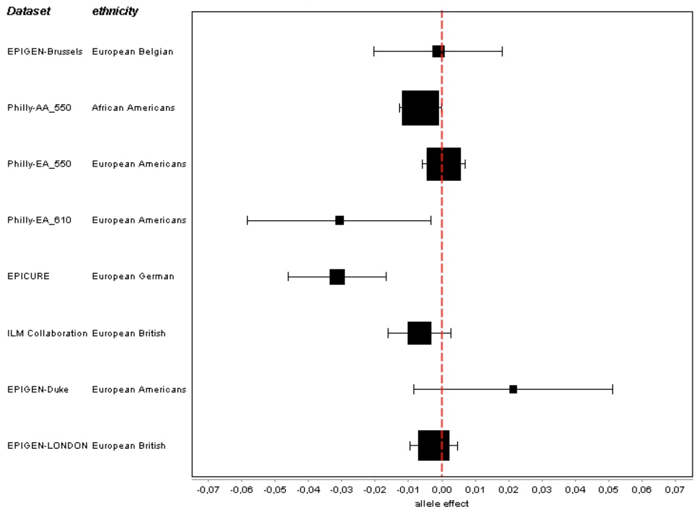

rs6732655 – Focal – meta p = $2.03x10^{-6}$

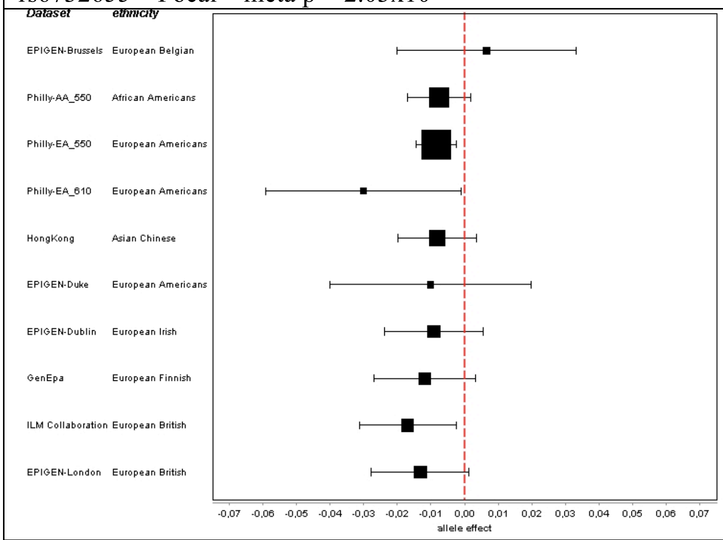

Supplementary Figure 5. Logistic regression. Gender and the first 20 PCAs were included as covariates in the logistic regression.

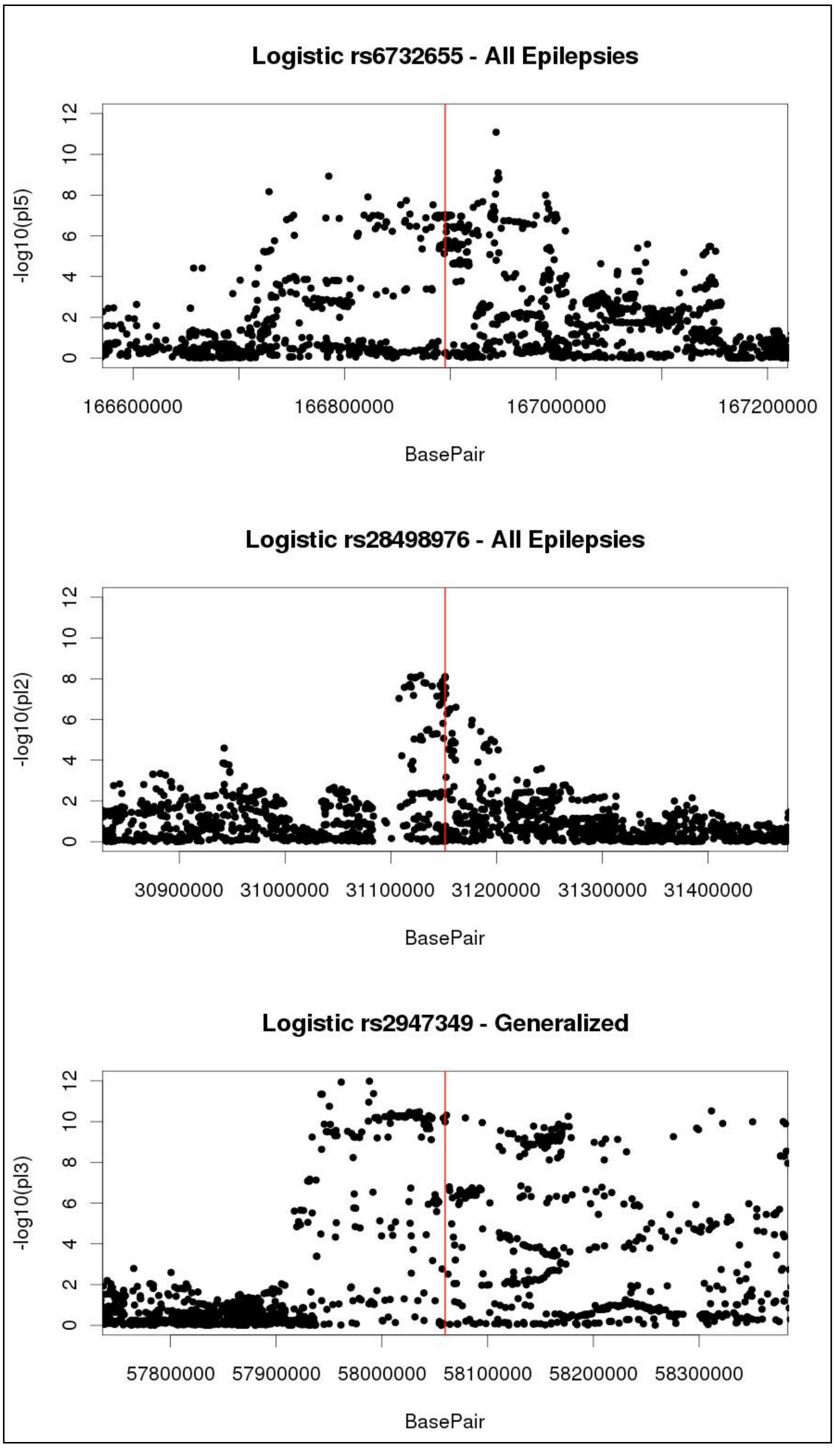

Supplementary Figure 6. Conditional analysis. Gender included as co-variate.

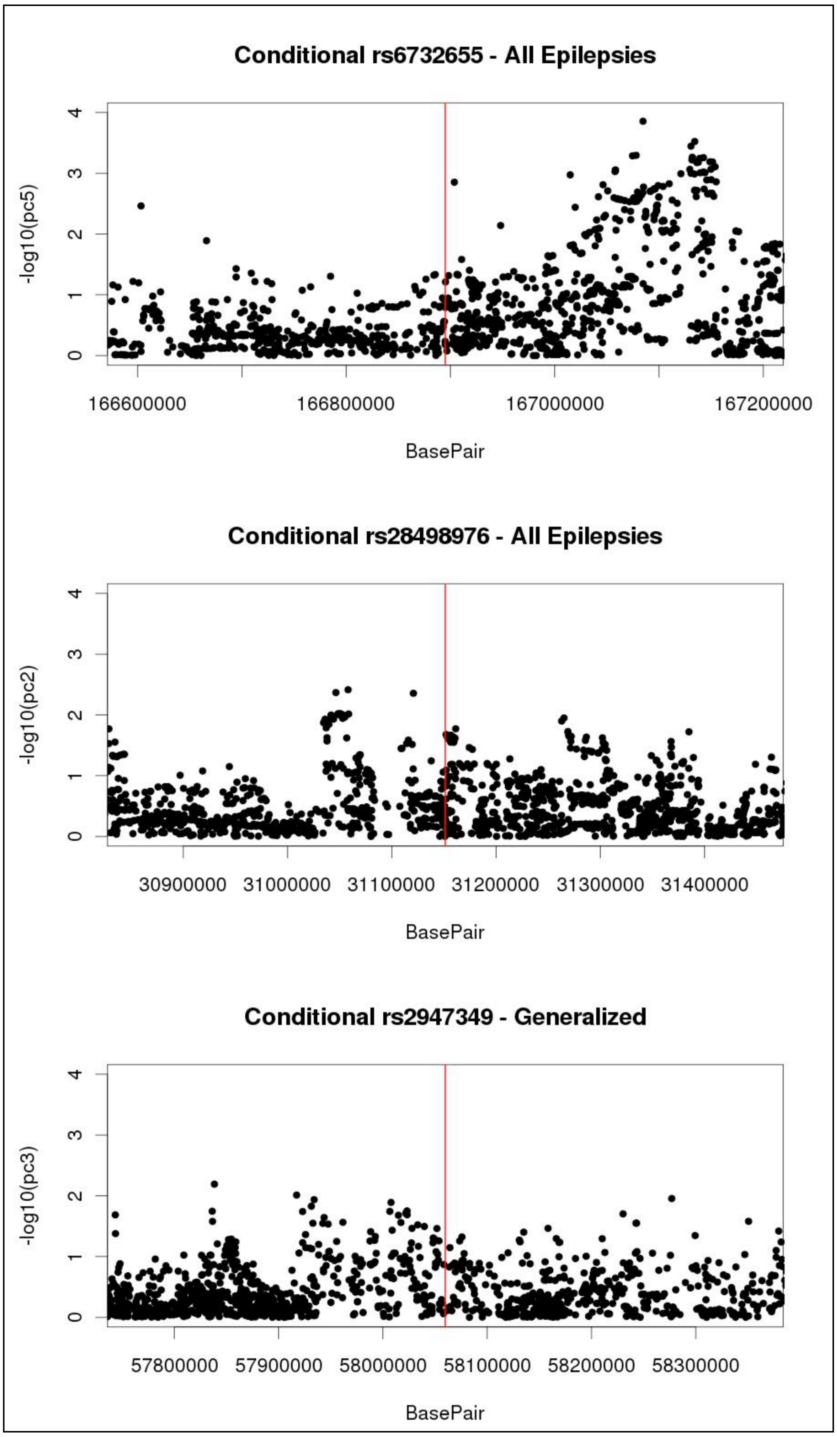

Supplementary Figure 7: Magnitude and direction of rs28498976 (*PCDH7*) across 3 phenotypes.

rs28498976 – All epilepsy – meta p = $5.44x10^{-9}$

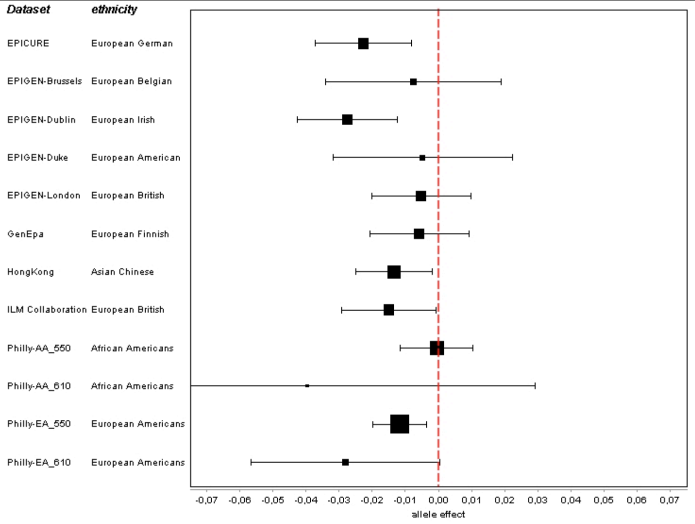

rs28498976 – GGE – meta p = $1.39x10^{-6}$

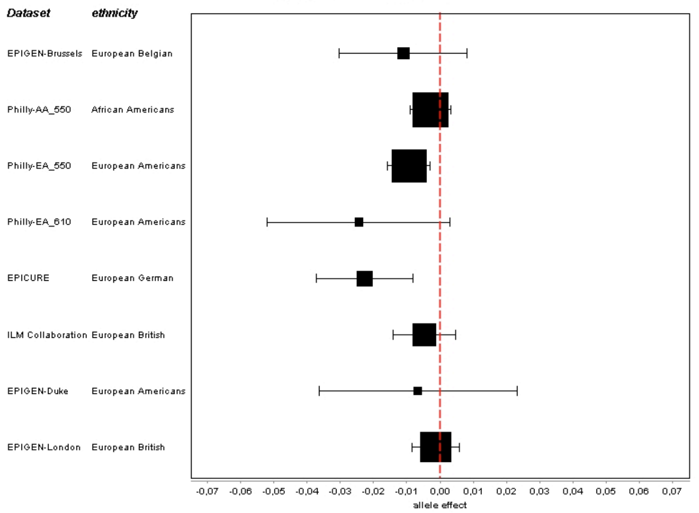

rs28498976 – Focal – meta p = 0.0014

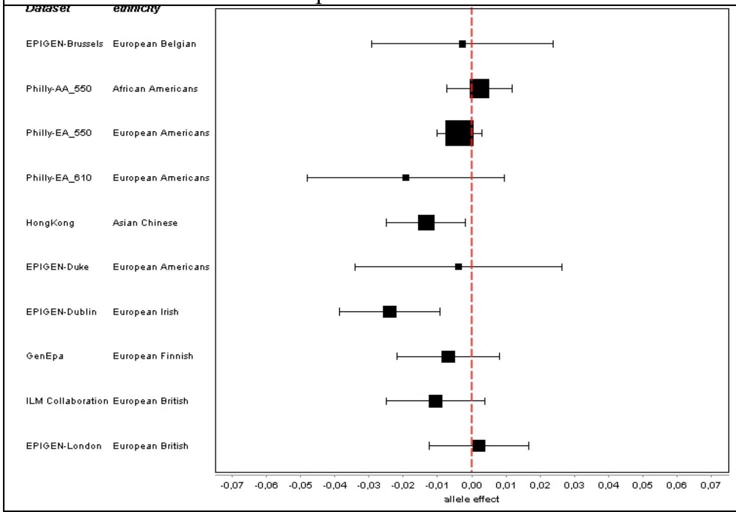

Supplementary Figure 8: Sub-threshold signal at 3q26.2 (all epilepsy).

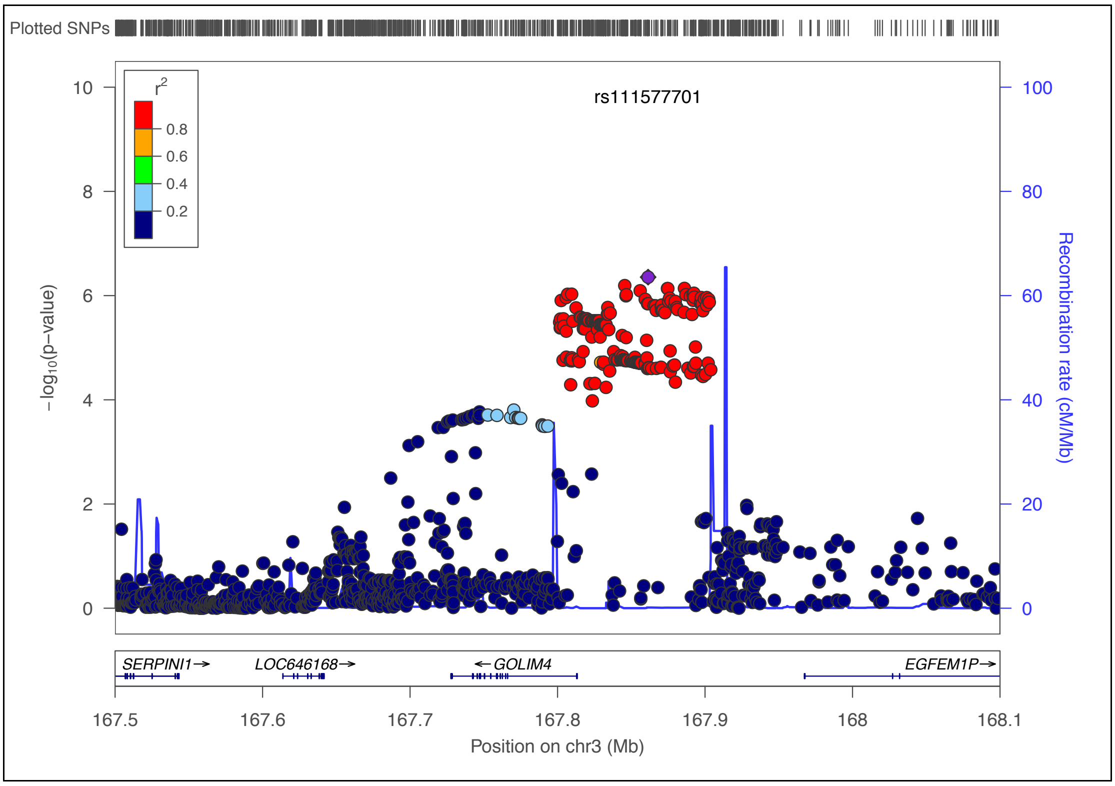

Supplementary Figure 9: Sub-threshold signal at 4p12 (all epilepsy).

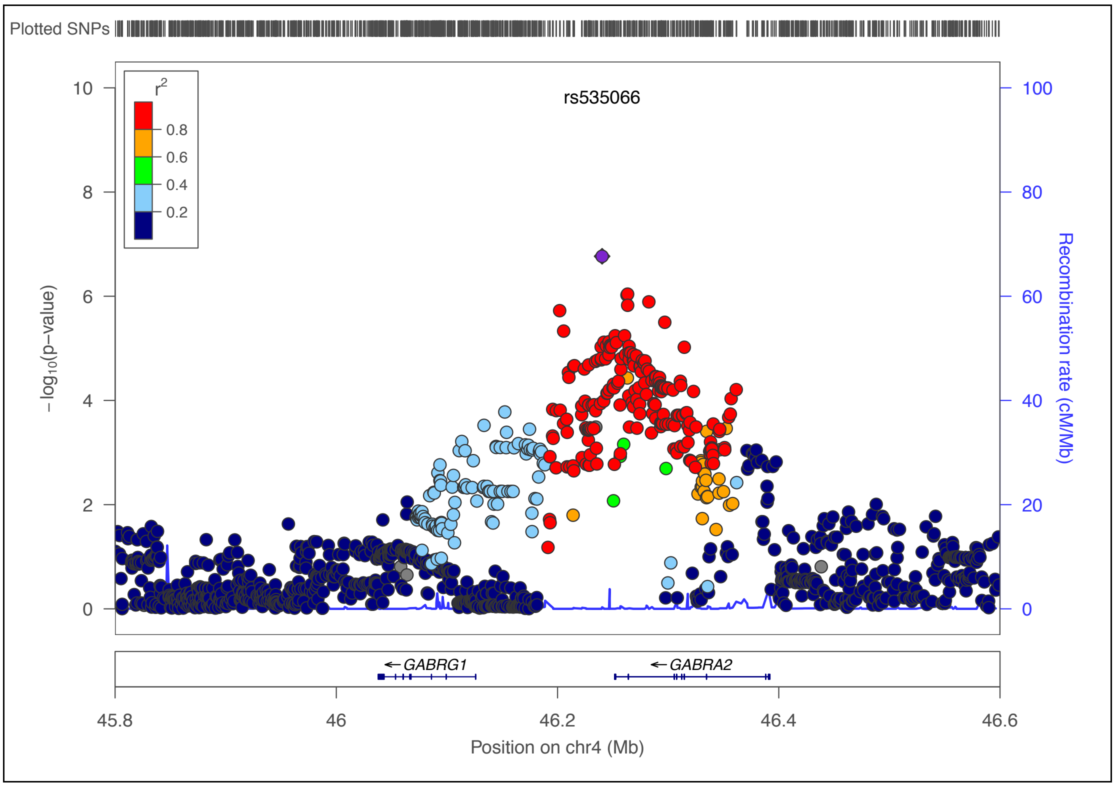

Supplementary Figure 10: Magnitude and direction of rs2947349 (*VRK2*) across 3 phenotypes.

rs2947349 – GGE – meta p = 9.99×10^{-9}

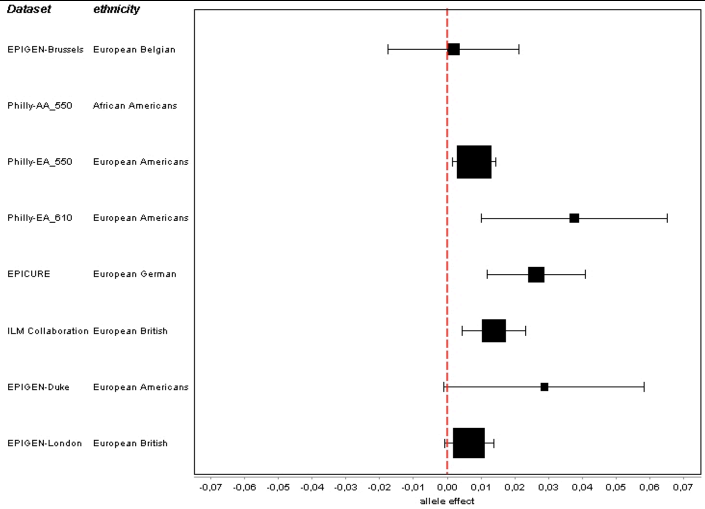

rs2947349 – All epilepsy – meta p = 5.34×10^{-5}

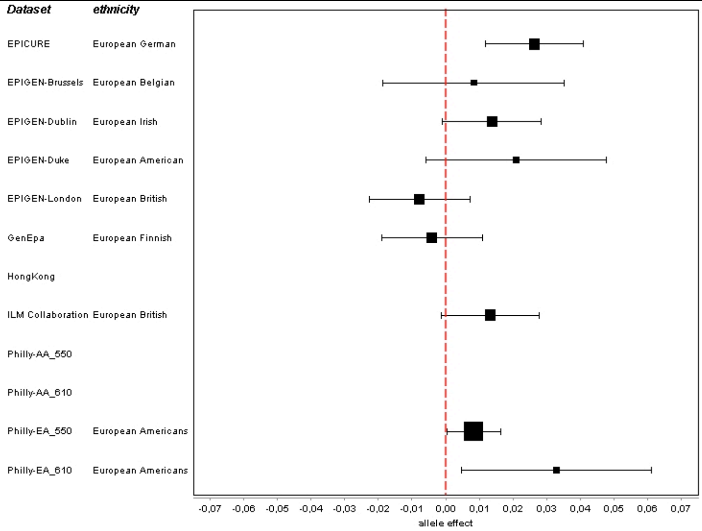

rs2947349 – Focal – meta p = 0.32

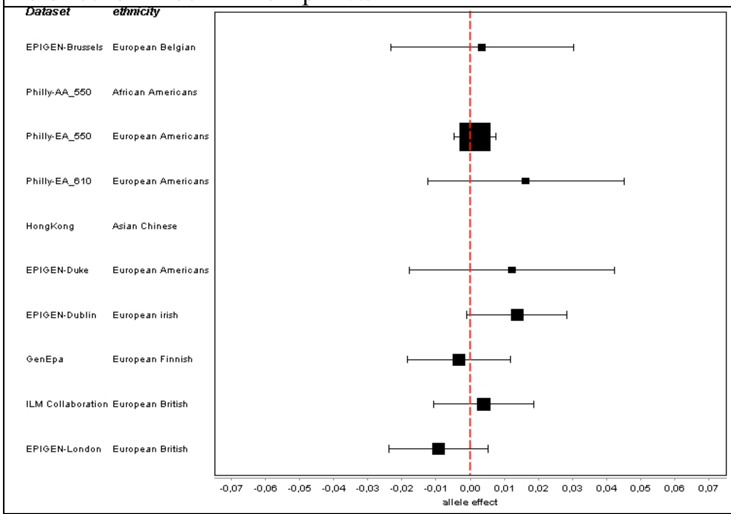

Supplementary Figure 11: Sub-threshold signal at 4p15.1 (GGE).

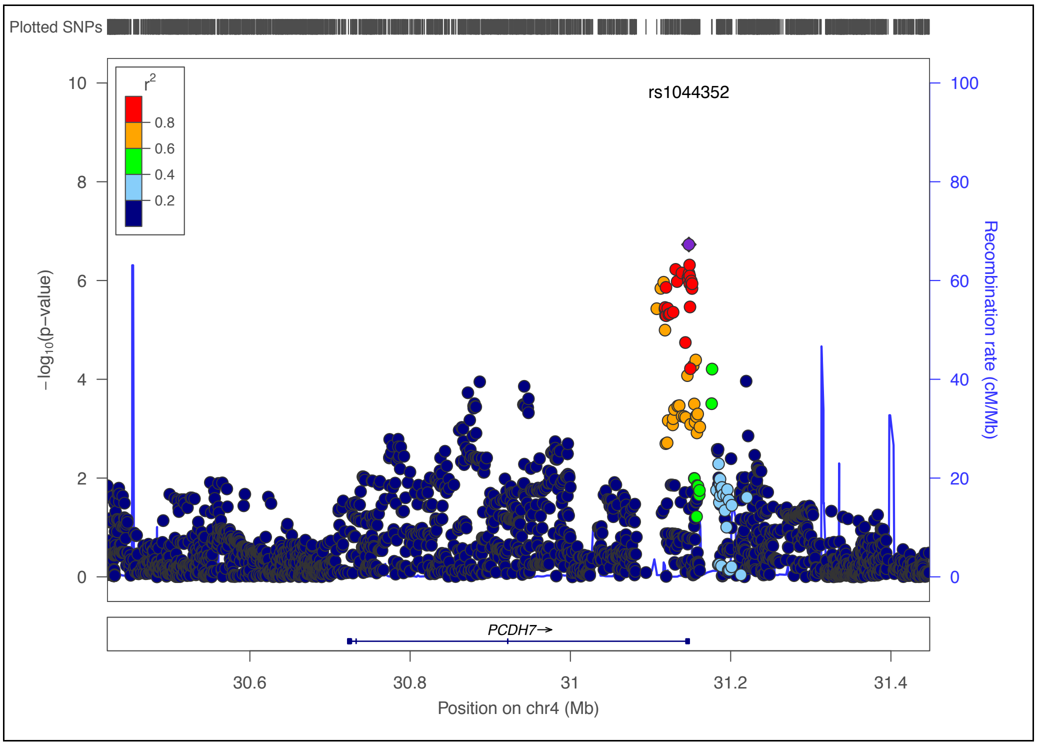

Supplementary Figure 12: Sub-threshold signal at 5q22.3 (GGE).

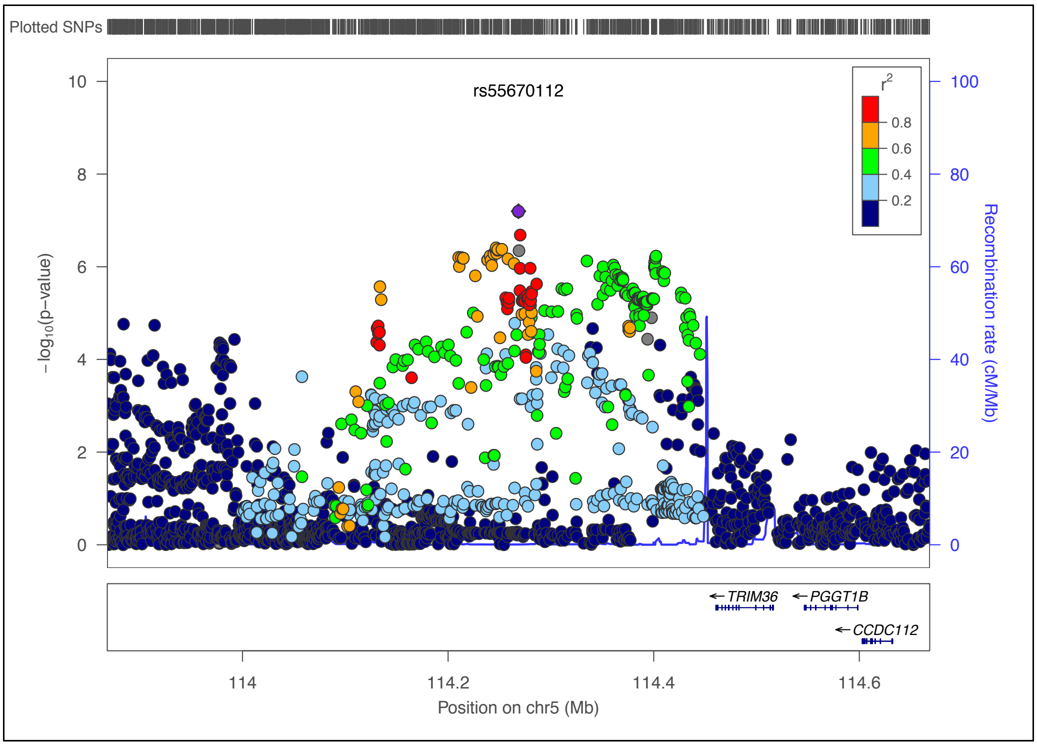

Supplementary Figure 13: Sub-threshold signal at 11q22.2 (GGE).

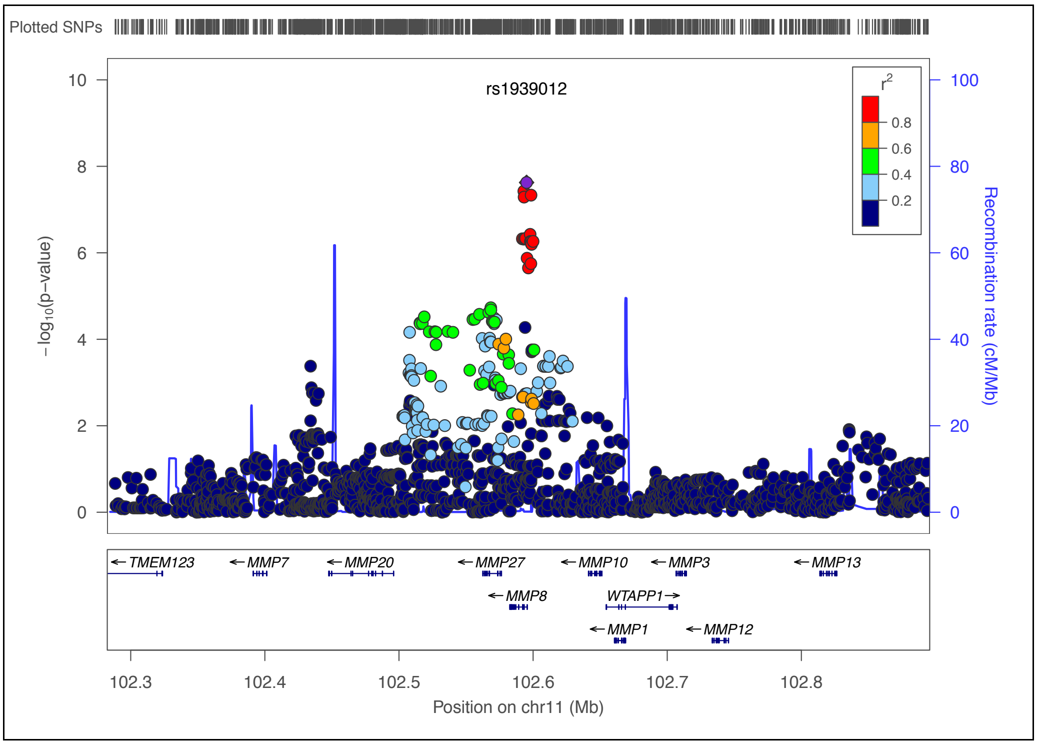

Supplementary Figure 14: Magnitude and direction of rs1939012 (*MMP8*) across 3 phenotypes.

rs1939012 – GGE – meta p = 2.37×10^{-8}

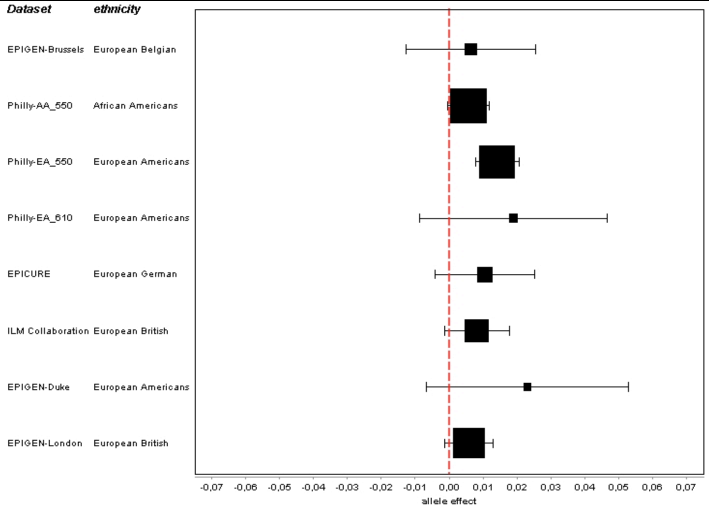

rs1939012 – All epilepsy – meta p = 0.02

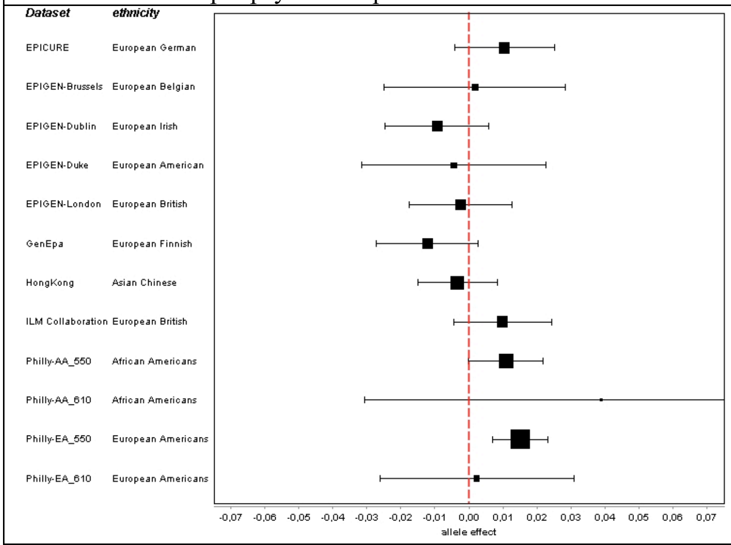

rs1939012 – Focal – meta p = 0.89

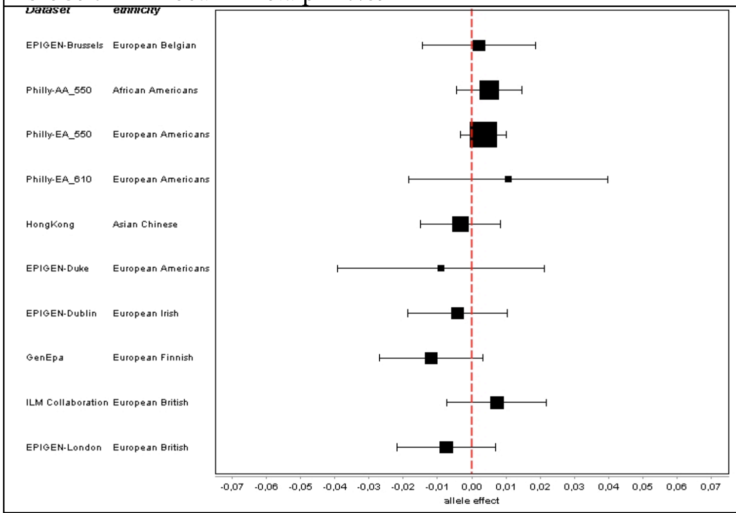

Supplementary Figure 15: Sub-threshold signal at 2q24.3 (focal).

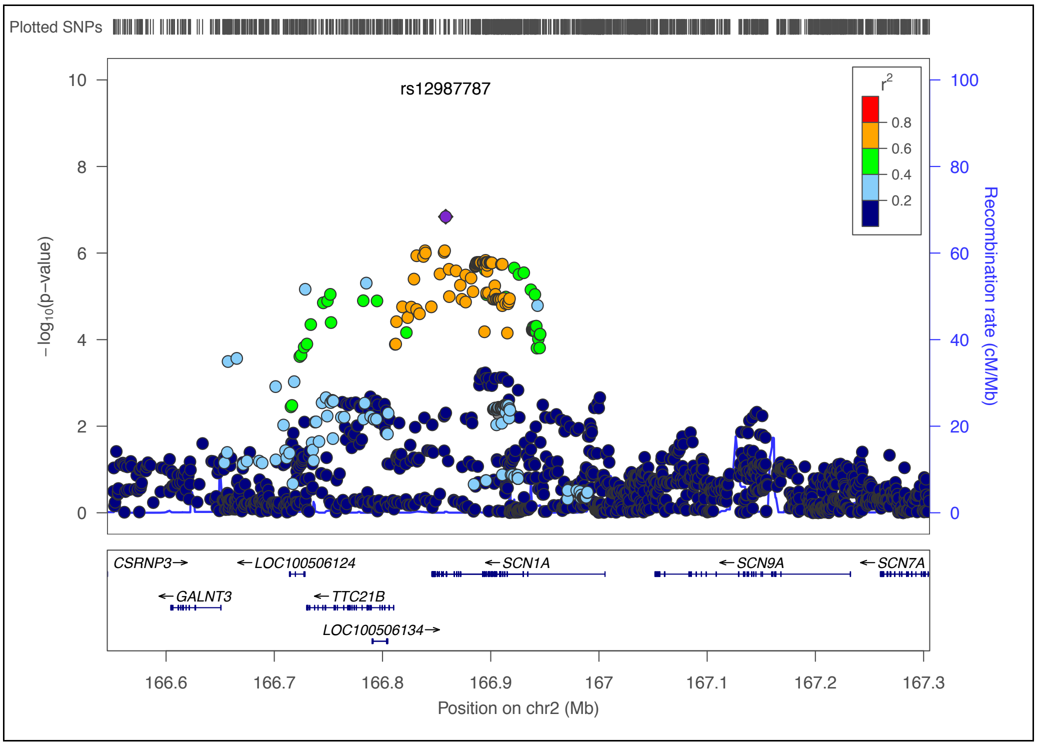

Supplementary Figure 16: Magnitude and direction of rs72823592 (17q21), previously reported to associate with GGE.

rs72823592– All epilepsy – meta p = 0.016

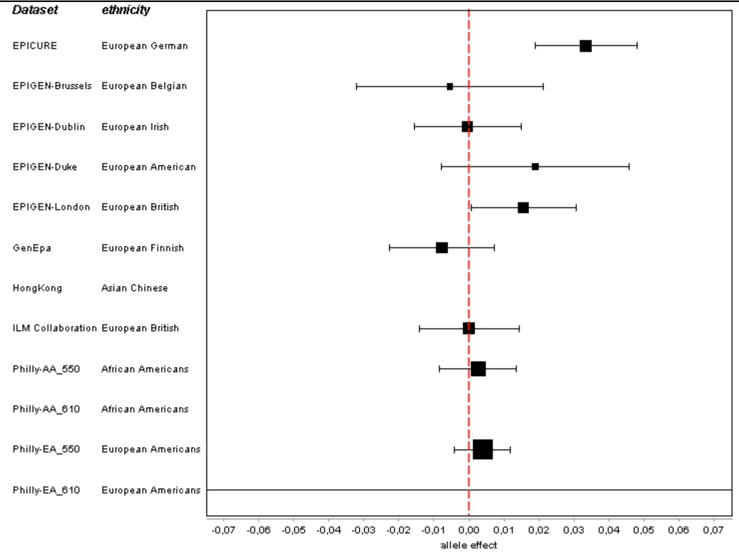

rs72823592– GGE – meta p = $7.91x10^{-4}$

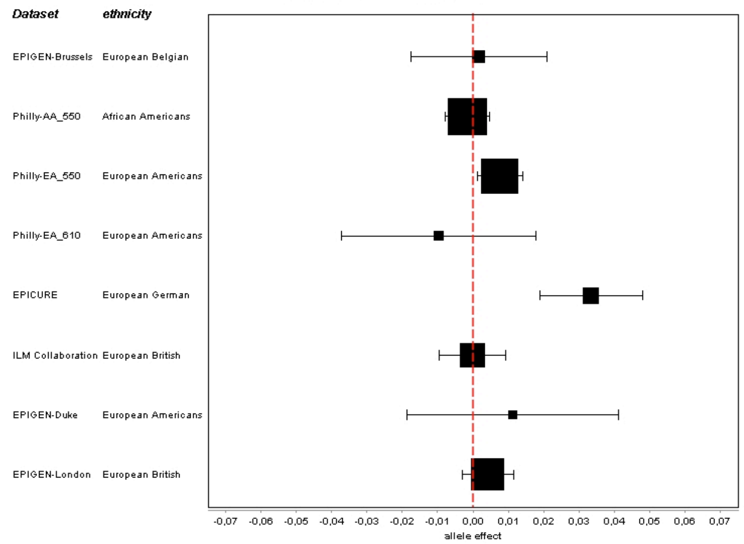

rs72823592– Focal – meta p = 0.93

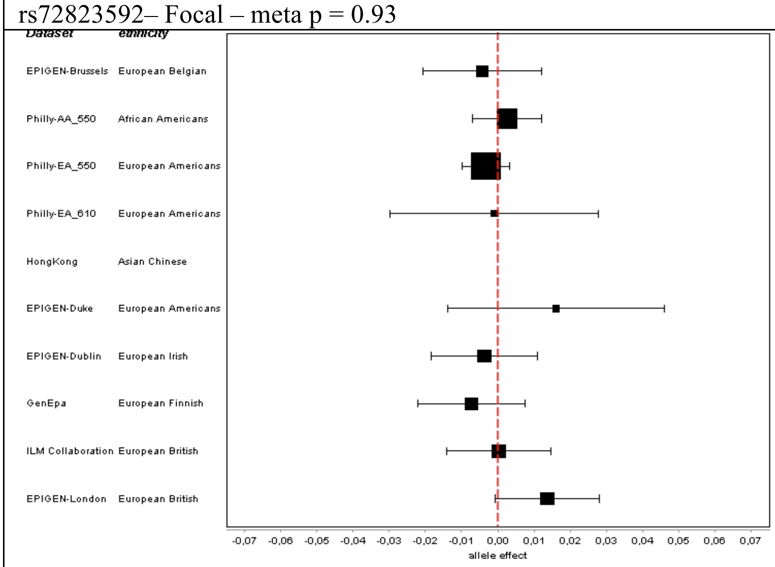

Supplementary Figure 17: Magnitude and direction of rs7587026 (*SCN1A*), previously reported to associate with mTLE+HS.

rs7587026 – All epilepsy – meta p = 1.14x10^{-5}

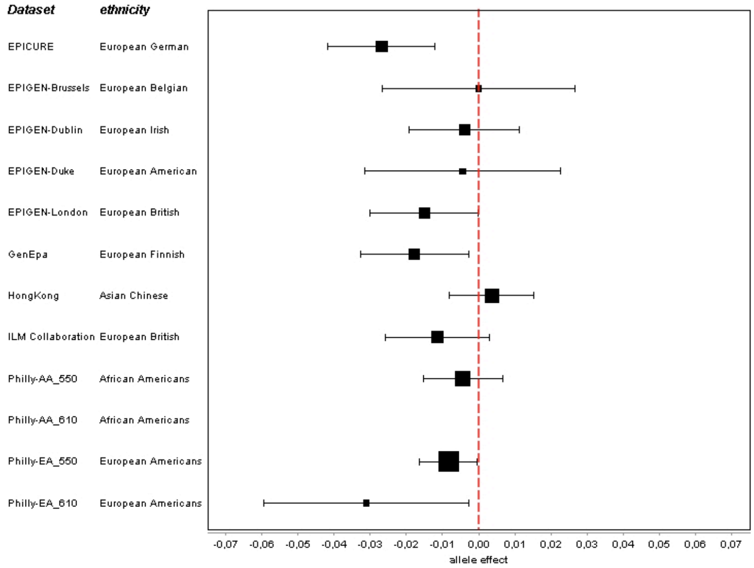

rs7587026 – GGE – meta p = 2.08x10^{-4}

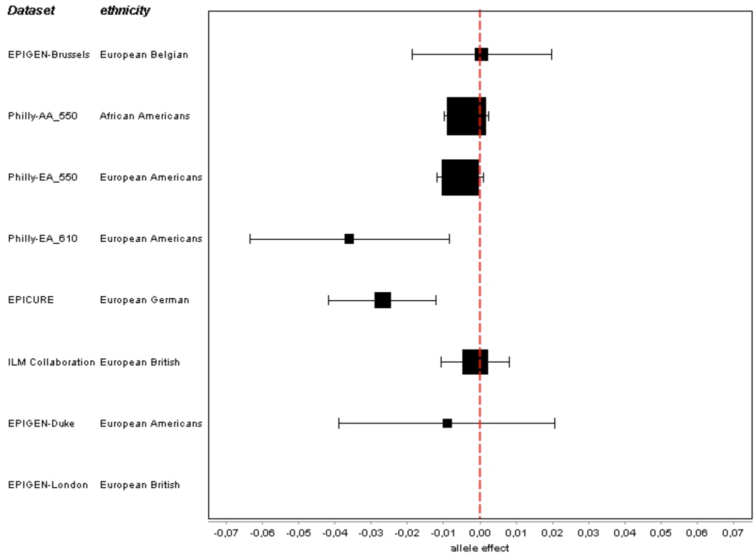

rs7587026 – Focal – meta p = 0.013

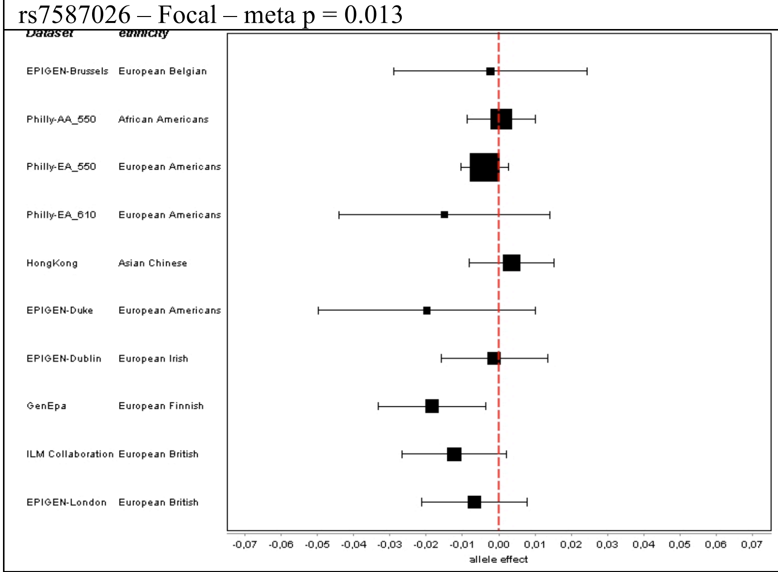

Supplementary Figure 18: Magnitude and direction of rs2292096 (*CAMSAP1L1*), previously reported to associate with focal epilepsy.

rs2292096 – All epilepsy – meta p = 0.049

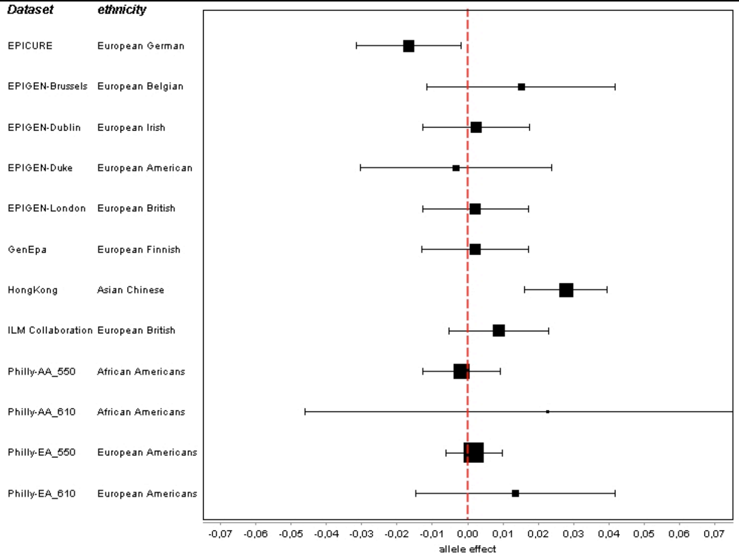

rs2292096 – GGE – meta p = 0.057

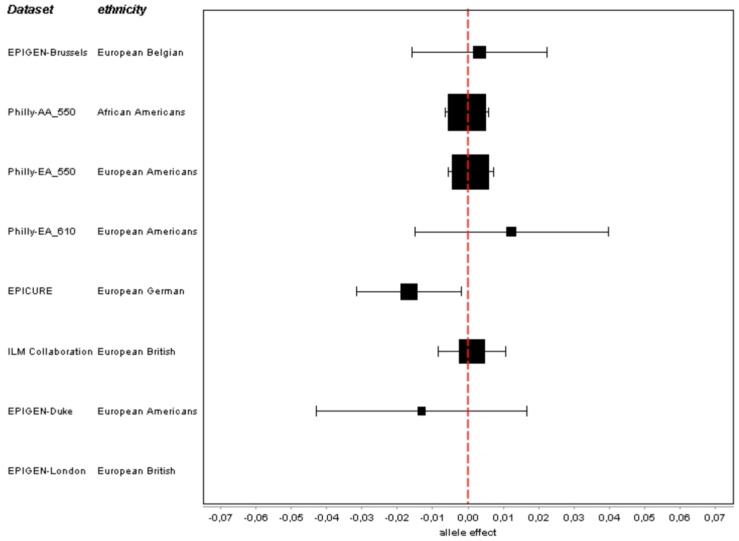

rs2292096 – Focal – meta p = 0.0032

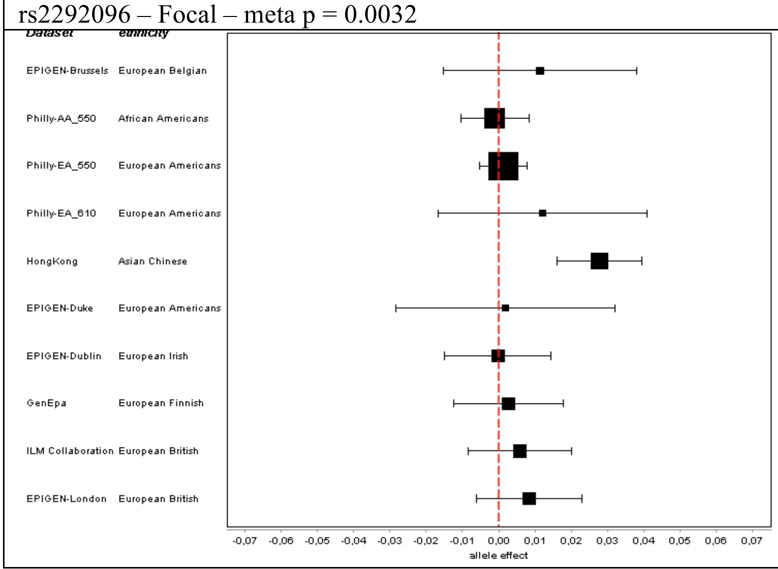

SUPPLEMENTARY TABLES

Supplementary Table 1: Details of pre-quality control numbers for individual case and control cohort.

Index GWAS	Ethnicity[1]	Epilepsy cases	GGE	Focal	Population controls[3]
EPIGEN-Dublin	Irish	650	16	532	2232
EPIGEN-Brussels	Belgian	564	60	429	1689
EPIGEN-Duke	AA[2] & EA	1250	173	880	527
EPIGEN-London	British + other	1326	109	910	2501
ILM Collaboration	European-cauc	1865	245	1350	2699
GenEpa	Finnish	422	-	422	1963
EPICURE	NW- European	1523	1443	-	2454
Philadelphia[2,4]	Various	1960	946	1004	13160
Hong Kong	Asian-Han	504	-	504	3500
**TOTAL**		**10064**	**2992**	**6031**	**30725**

[1]Broad ethnicity of the cohort. AA: African American. EA: European American. Other: indicates mixed, as would be expected in a cosmopolitan population. European-cauc: European-Caucasian. NW-European: North-West European. [2]EPIGEN-Duke individuals of AA ancestry were (post-QC) merged with Philadelphia_550_AA cohort. [3]See Supplementary Table 2 for further details on control cohorts. [4]The Philadelphia cohort was split into 4 post quality control groups, based on ancestry and genotyping platform.

Supplementary Table 2: Control cohorts.

Contributor	Ancestry	Control numbers	Platform	Chip
Trinity Student Study[a]	Irish	2232	Illumina	Omni1-Quad
Belgian donors[b]	Belgian	1000	Illumina	300 V1 & V2
ALS study[c]	US-Cauc	527	Illumina	550, 300, 610, iselect-Quad
National Blood Bank Service[d]	British	2501	Illumina	1.2M
Wellcome 1958 Birth Cohort[e]	British	2699	Illumina	1.2M
GenEpa[f]	Finnish	287	Illumina	610
HSBC controls	Finnish	1676	Illumina	610
KORA & PopGen[g]	NW-European	2500	Affymetrix	6.0
CHOP/CAG controls	US (AA&Cauc)	13160	Illumina	550, 610, Omni-Express
Hong Kong[h]	Han	3500	Illumina	550, 610

[a]Healthy young adult volunteers of Irish ancestry (age between 18 and 28 years)[9,10]. [b]Blood donors and healthy volunteers of Belgian ancestry[11]. [c]Controls were obtained from previous studies of Amyotrophic Lateral Sclerosis via the database of Genotypes and Phenotypes (dbGaP phs000101.v2.p1). [d]The National Blood Service and [e]UK1958 Birth Cohort controls were typed at the Wellcome Trust Sanger Institute (UK) using an Illumina Human1.2M custom array. [f]Individuals of Finnish origin, screened for neurological conditions, recruited as control subjects. [g]German control subjects were obtained from the PopGen biobank and the KORA (Cooperative Health Research in the Region of Augsburg) research platform representing epidemiologically recruited cohorts from the Northern (Schleswig, PopGen) and Southern (Augsburg, KORA) regions of Germany. [h]Subjects from other studies in Hong Kong and from healthy controls in Taiwan. For all samples, we tested for and removed any duplicates.

Supplementary Table 3: Confirmatory genotyping.

	Caucasian				African-American		Asian-Han	TOTAL
	*Brussels	*Dublin	*London	EPICURE	Philadelphia	*Duke	Hong Kong	combined
rs6732655	30/30<br>(1)	24/24<br>(1)	49/50<br>(0.98)	20/20<br>(1)	150/150<br>(1)	-	50/50<br>(1)	324/325<br>(0.99)
rs28498976	30/33<br>(0.91)	72/74<br>(0.97)	68/68<br>(1)	31/31<br>(1)	92/92 (1)	134/140<br>(0.96)	217/218 (0.99)	644/656<br>(0.98)
rs2947349	33/33<br>(1)	57/58<br>(0.98)	32/32<br>(1)	33/33<br>(1)	-	-	34/37<br>(0.92)	189/193 (0.98)

*EPIGEN cohort

Supplementary Table 4 A/B/C: Results of enrichment analysis for (a) all epilepsy, (b) GGE and (c) focal and phenotypes.

Table legend: GENE_SET_ID: Gene Ontology (GO) pathway. N_GENES_IN_SET: Number of genes in that GO pathway. N_ASSOC_INT_IN_SET: Number of genes from associated intervals within that GO pathway. ASSOC_GENES_IN_INT: The names of the genes in interval from that GO pathway. EMPIRICAL_P: empirical p value. CORRECTED_P: P value corrected for number of gene pathways considered.

A – all epilepsy

GENE_SET_ID	N_GENES_IN_SET	N_ASSOC_INT_IN_SET	ASSOC_GENES_IN_INT	EMPIRICAL_P	CORRECTED_P
GO:0005164 Tumor necrosis factor receptor binding	25	2	BRE STAT1	0.00119976	0.623688
GO:0014047 Glutamate secretion	18	2	GLS RIMS1	0.00159968	0.696652
GO:0018024 Histone-lysine N-methyltransferase activity	41	2	DOT1L PRDM2	0.00179964	0.722139
GO:0042054 Histone methyltransferase activity	50	2	DOT1L PRDM2	0.00239952	0.787106
GO:0032813 Tumor necrosis factor receptor superfamily binding	36	2	BRE STAT1	0.00239952	0.787106
GO:0016278 Lysine N-methyltransferase activity	47	2	DOT1L PRDM2	0.00259948	0.806097
GO:0016279 Protein-lysine N-methyltransferase activity	47	2	DOT1L PRDM2	0.00259948	0.806097
GO:0018022 Peptidyl-lysine methylation	57	2	DOT1L PRDM2	0.00319936	0.845577
GO:0017156 Calcium ion-dependent exocytosis	29	2	RIMS1 SPESP1	0.00319936	0.845577
GO:0005801 Cis-Golgi network	29	2	COPZ2 GOLIM4	0.00359928	0.874563
GO:0046148 Pigment biosynthetic process	46	2	AP3D1 NFE2L1	0.0039992	0.887056
GO:0034968 Histone lysine methylation	57	2	DOT1L PRDM2	0.00419916	0.895552
GO:0008170 N-methyltransferase activity	69	2	DOT1L PRDM2	0.00479904	0.912044
GO:0042440 Pigment metabolic process	55	2	AP3D1 NFE2L1	0.00539892	0.923038
GO:0016571 Histone methylation	70	2	DOT1L PRDM2	0.00619876	0.935532
GO:0008276 Protein methyltransferase activity	70	2	DOT1L PRDM2	0.00639872	0.94003
GO:0008757 S-adenosylmethionine-dependent methyltransferase activity	107	2	DOT1L PRDM2	0.00879824	0.967516
GO:0006944 Cellular membrane fusion	71	2	RIMS1 SPESP1	0.0089982	0.970015

GO:0061025 Membrane fusion	75	2	RIMS1 SPESP1	0.0089982	0.970015
GO:0006835 Dicarboxylic acid transport	57	2	GLS RIMS1	0.00919816	0.972014
GO:0048475 Coated membrane	76	2	AP3D1 COPZ2	0.0107978	0.981009
GO:0030117 Membrane coat	76	2	AP3D1 COPZ2	0.0107978	0.981009
GO:0001959 Regulation of cytokine-mediated signaling pathway	87	2	STAT1 VRK2	0.0113977	0.983508
GO:0003735 Structural constituent of ribosome	149	2	MRPL33 RPL10L	0.0125975	0.988006
GO:0008213 Protein alkylation	98	2	DOT1L PRDM2	0.0127974	0.989005
GO:0006479 Protein methylation	98	2	DOT1L PRDM2	0.0127974	0.989005
GO:0060759 Regulation of response to cytokine stimulus	93	2	STAT1 VRK2	0.015197	0.992004
GO:2000241 Regulation of reproductive process	93	2	ACVR1B NOX5	0.0173965	0.996002
GO:0048489 Synaptic vesicle transport	63	2	AP3D1 RIMS1	0.0179964	0.996502
GO:0097480 Establishment of synaptic vesicle localization	63	2	AP3D1 RIMS1	0.0179964	0.996502
GO:0097479 Synaptic vesicle localization	65	2	AP3D1 RIMS1	0.0223955	0.997501
GO:0042364 Water-soluble vitamin biosynthetic process	108	2	AP3D1 PNPO	0.0227954	0.997501
GO:0009110 Vitamin biosynthetic process	117	2	AP3D1 PNPO	0.0257948	0.997501
GO:0051650 Establishment of vesicle localization	113	2	AP3D1 RIMS1	0.029994	0.998501
GO:0043414 Macromolecule methylation	162	2	DOT1L PRDM2	0.0303939	0.998501
GO:0000790 Nuclear chromatin	156	2	CBX1 STAT1	0.0303939	0.998501
GO:0007269 Neurotransmitter secretion	75	2	GLS RIMS1	0.0311938	0.999
GO:0018205 Peptidyl-lysine modification	167	2	DOT1L PRDM2	0.0339932	1
GO:0012502 Induction of programmed cell death	157	2	NOX5 STAT1	0.034993	1
GO:0006917 Induction of apoptosis	157	2	NOX5 STAT1	0.034993	1
GO:0030574 Collagen catabolic process	70	2	MMP27 MMP8	0.0375925	1
GO:0044243 Multicellular organismal catabolic process	77	2	MMP27 MMP8	0.0381924	1
GO:0032963 Collagen metabolic process	80	2	MMP27 MMP8	0.0389922	1
GO:0044259 Multicellular organismal macromolecule	86	2	MMP27 MMP8	0.039992	1

metabolic process					
GO:0001505 Regulation of neurotransmitter levels	106	2	GLS RIMS1	0.0403919	1
GO:0051188 Cofactor biosynthetic process	125	2	NFE2L1 PNPO	0.0411918	1
GO:0044236 Multicellular organismal metabolic process	92	2	MMP27 MMP8	0.0411918	1
GO:0044389 Small conjugating protein ligase binding	151	2	ACVR1B FANCL	0.0435913	1
GO:0031625 Ubiquitin protein ligase binding	151	2	ACVR1B FANCL	0.0435913	1
GO:0004222 Metalloendopeptidase activity	103	2	MMP27 MMP8	0.0445911	1
GO:0051648 Vesicle localization	139	2	AP3D1 RIMS1	0.0479904	1

B - GGE

GENE_SET_ID	N_GENES_IN_SET	N_ASSOC_INT_IN_SET	ASSOC_GENES_IN_INT	EMPIRICAL_P	CORRECTED_P
GO:0005164 Tumor necrosis factor receptor binding	25	2	BRE STAT1	0.00119976	0.623688
GO:0014047 Glutamate secretion	18	2	GLS RIMS1	0.00159968	0.696652
GO:0018024 Histone-lysine N-methyltransferase activity	41	2	DOT1L PRDM2	0.00179964	0.722139
GO:0042054 Histone methyltransferase activity	50	2	DOT1L PRDM2	0.00239952	0.787106
GO:0032813 Tumor necrosis factor receptor superfamily binding	36	2	BRE STAT1	0.00239952	0.787106
GO:0016278 Lysine N-methyltransferase activity	47	2	DOT1L PRDM2	0.00259948	0.806097
GO:0016279 Protein-lysine N-methyltransferase activity	47	2	DOT1L PRDM2	0.00259948	0.806097
GO:0018022 Peptidyl-lysine methylation	57	2	DOT1L PRDM2	0.00319936	0.845577
GO:0017156 Calcium ion-dependent exocytosis	29	2	RIMS1 SPESP1	0.00319936	0.845577
GO:0005801 Cis-Golgi network	29	2	COPZ2 GOLIM4	0.00359928	0.874563
GO:0046148 Pigment biosynthetic process	46	2	AP3D1 NFE2L1	0.0039992	0.887056
GO:0034968 Histone lysine methylation	57	2	DOT1L PRDM2	0.00419916	0.895552
GO:0008170 N-methyltransferase activity	69	2	DOT1L PRDM2	0.00479904	0.912044
GO:0042440 Pigment metabolic process	55	2	AP3D1 NFE2L1	0.00539892	0.923038
GO:0016571 Histone methylation	70	2	DOT1L PRDM2	0.00619876	0.935532

GO:0008276 Protein methyltransferase activity	70	2	DOT1L PRDM2	0.00639872	0.94003
GO:0008757 S-adenosylmethionine-dependent methyltransferase activity	107	2	DOT1L PRDM2	0.00879824	0.967516
GO:0006944 Cellular membrane fusion	71	2	RIMS1 SPESP1	0.0089982	0.970015
GO:0061025 Membrane fusion	75	2	RIMS1 SPESP1	0.0089982	0.970015
GO:0006835 Dicarboxylic acid transport	57	2	GLS RIMS1	0.00919816	0.972014
GO:0048475 Coated membrane	76	2	AP3D1 COPZ2	0.0107978	0.981009
GO:0030117 Membrane coat	76	2	AP3D1 COPZ2	0.0107978	0.981009
GO:0001959 Regulation of cytokine-mediated signaling pathway	87	2	STAT1 VRK2	0.0113977	0.983508
GO:0003735 Structural constituent of ribosome	149	2	MRPL33 RPL10L	0.0125975	0.988006
GO:0008213 Protein alkylation	98	2	DOT1L PRDM2	0.0127974	0.989005
GO:0006479 Protein methylation	98	2	DOT1L PRDM2	0.0127974	0.989005
GO:0060759 Regulation of response to cytokine stimulus	93	2	STAT1 VRK2	0.015197	0.992004
GO:2000241 Regulation of reproductive process	93	2	ACVR1B NOX5	0.0173965	0.996002
GO:0048489 Synaptic vesicle transport	63	2	AP3D1 RIMS1	0.0179964	0.996502
GO:0097480 Establishment of synaptic vesicle localization	63	2	AP3D1 RIMS1	0.0179964	0.996502
GO:0097479 Synaptic vesicle localization	65	2	AP3D1 RIMS1	0.0223955	0.997501
GO:0042364 Water-soluble vitamin biosynthetic process	108	2	AP3D1 PNPO	0.0227954	0.997501
GO:0009110 Vitamin biosynthetic process	117	2	AP3D1 PNPO	0.0257948	0.997501
GO:0051650 Establishment of vesicle localization	113	2	AP3D1 RIMS1	0.029994	0.998501
GO:0043414 Macromolecule methylation	162	2	DOT1L PRDM2	0.0303939	0.998501
GO:0000790 Nuclear chromatin	156	2	CBX1 STAT1	0.0303939	0.998501
GO:0007269 Neurotransmitter secretion	75	2	GLS RIMS1	0.0311938	0.999
GO:0018205 Peptidyl-lysine modification	167	2	DOT1L PRDM2	0.0339932	1
GO:0012502 Induction of programmed cell death	157	2	NOX5 STAT1	0.034993	1
GO:0006917 Induction of apoptosis	157	2	NOX5 STAT1	0.034993	1

GO:0030574 Collagen catabolic process	70	2	MMP27 MMP8	0.0375925	1
GO:0044243 Multicellular organismal catabolic process	77	2	MMP27 MMP8	0.0381924	1
GO:0032963 Collagen metabolic process	80	2	MMP27 MMP8	0.0389922	1
GO:0044259 Multicellular organismal macromolecule metabolic process	86	2	MMP27 MMP8	0.039992	1
GO:0001505 Regulation of neurotransmitter levels	106	2	GLS RIMS1	0.0403919	1
GO:0051188 Cofactor biosynthetic process	125	2	NFE2L1 PNPO	0.0411918	1
GO:0044236 Multicellular organismal metabolic process	92	2	MMP27 MMP8	0.0411918	1
GO:0044389 Small conjugating protein ligase binding	151	2	ACVR1B FANCL	0.0435913	1
GO:0031625 Ubiquitin protein ligase binding	151	2	ACVR1B FANCL	0.0435913	1
GO:0004222 Metalloendopeptidase activity	103	2	MMP27 MMP8	0.0445911	1
GO:0051648 Vesicle localization	139	2	AP3D1 RIMS1	0.0479904	1

C– focal

GENE_SET_ID	N_GENES_IN_SET	N_ASSOC_INT_IN_SET	ASSOC_GENES_IN_INT	EMPIRICAL_P	CORRECTED_P
GO:0043235 Receptor complex	184	3	CACNG5 GHR KCTD8	0.00479904	0.769115
GO:0015081 Sodium ion transmembrane transporter activity	122	2	SCN1A SLC13A4	0.0123975	0.914043
GO:0035725 Sodium ion transmembrane transport	124	2	SCN1A SLC13A4	0.0137972	0.927036
GO:0045211 Postsynaptic membrane	188	3	CACNG5 KCTD8 TENM2	0.0191962	0.952024
GO:0006814 Sodium ion transport	166	2	SCN1A SLC13A4	0.0231954	0.963018
GO:0022843 Voltage-gated cation channel activity	130	2	CACNG5 SCN1A	0.0313937	0.972014
GO:0005244 Voltage-gated ion channel activity	174	2	CACNG5 SCN1A	0.0413917	0.976512
GO:0022832 Voltage-gated channel activity	174	2	CACNG5 SCN1A	0.0413917	0.976512

Supplementary Table 5: Minimum p values for susceptibility loci (p<5x10-8) with outcome of newly treated epilepsy using data from Speed et al., 2014.[3] We considered both the index SNP (Table 2) and all SNPs within a 20Kb window around each of the 5 genes (*SCN1A*, *PCDH7*, *VRK2*/*FANCL*, *MMP8*). Analyses were conducted before (A) and after (B) adjusting for clinical prognostic factors. Min Pvalue refers to the minimum p value of association with outcome of newly treated epilepsy for any SNP in the region; Min Pvalue BF is the minimum p value Bonferroni (BF) corrected for number of SNPs in that gene.

A. With clinical covariates

Gene_	Chr	Min Pvalue	Min Pvalue BF
VRK2	2	3.97e-02	1.00e+00
FANCL	2	3.99e-02	1.00e+00
SCN1A	2	3.51e-02	1.00e+00
PCDH7	4	1.32e-03	7.96e-01
MMP8	11	8.14e-04	1.83e-01

B. Without clinical covariates

Gene_	Chr	Min Pvalue	Min Pvalue BF
VRK2	2	2.35e-02	9.92e-01
FANCL	2	2.19e-02	9.96e-01
SCN1A	2	4.61e-02	1.00e+00
PCDH7	4	2.55e-03	9.53e-01
MMP8	11	3.55e-03	5.86e-01

SUPPLEMENTARY REFERENCES

1. EPICURE Consortium, EMINet Consortium, Steffens M, et al. Genome-wide association analysis of genetic generalized epilepsies implicates susceptibility loci at 1q43, 2p16.1, 2q22.3 and 17q21.32. *Hum Mol Genet* 2012; **21**(24): 5359-72.
2. Buono RJ, Lohoff FW, Sander T, et al. Association between variation in the human KCNJ10 potassium ion channel gene and seizure susceptibility. *Epilepsy Res* 2004; **58**(2-3): 175-83.
3. Speed D, Hoggart C, Petrovski S, et al. A genome-wide association study and biological pathway analysis of epilepsy prognosis in a prospective cohort of newly treated epilepsy. *Hum Mol Genet* 2014; **23**: 247-258.
4. Howie BN, Donnelly P, Marchini J. A flexible and accurate genotype imputation method for the next generation of genome-wide association studies. *PLoS Genet* 2009; **5**(6): e1000529.
5. Lippert C, Listgarten J, Liu Y, Kadie CM, Davidson RI, Heckerman D. FaST linear mixed models for genome-wide association studies. *Nat Methods* 2011; **8**(10): 833-5.
6. Willer CJ, Li Y, Abecasis GR. METAL: fast and efficient meta-analysis of genomewide association scans. *Bioinformatics* 2010; **26**(17): 2190-1.
7. Dempster ER, Lerner IM. Heritability of Threshold Characters. *Genetics* 1950; **35**(2): 212-36.
8. Lee PH, O'Dushlaine C, Thomas B, Purcell SM. INRICH: interval-based enrichment analysis for genome-wide association studies. *Bioinformatics* 2012; **28**(13): 1797-9.
9. Desch KC, Ozel AB, Siemieniak D, et al. Linkage analysis identifies a locus for plasma von Willebrand factor undetected by genome-wide association. *Proc Natl Acad Sci U S A* 2013; **110**(2): 588-93.
10. Mills JL, Carter TC, Scott JM, et al. Do high blood folate concentrations exacerbate metabolic abnormalities in people with low vitamin B-12 status? *Am J Clin Nutr* 2011; **94**(2): 495-500.
11. Libioulle C, Louis E, Hansoul S, et al. Novel Crohn disease locus identified by genome-wide association maps to a gene desert on 5p13.1 and modulates expression of PTGER4. *PLoS Genet* 2007; **3**(4): e58.
